# Reconstituting *Drosophila* Centromere Identity in Human Cells

**DOI:** 10.1016/j.celrep.2019.08.067

**Published:** 2019-10-08

**Authors:** Virginie Roure, Bethan Medina-Pritchard, Vasiliki Lazou, Luciano Rago, Eduard Anselm, Daniela Venegas, A. Arockia Jeyaprakash, Patrick Heun

**Affiliations:** 1 Wellcome Centre for Cell Biology, Institute of Cell Biology, University of Edinburgh, Edinburgh EH9 3QR, UK; 2 Max-Planck-Institute of Immunobiology, Stübeweg 51, 79108 Freiburg, Germany

**Keywords:** epigenetics, centromere, chromatin, chromosomes, CENP-A, CENP-C, CAL1

## Abstract

The centromere is an essential chromosomal region required for accurate chromosome segregation. Most eukaryotic centromeres are defined epigenetically by the histone H3 variant, centromere protein (CENP)-A, yet how its self-propagation is achieved remains poorly understood. Here, we develop a heterologous system to reconstitute epigenetic inheritance of centromeric chromatin by ectopically targeting the *Drosophila* centromere proteins dCENP-A, dCENP-C, and CAL1 to LacO arrays in human cells. Dissecting the function of these three components uncovers the key role of self-association of dCENP-C and CAL1 for their mutual interaction and dCENP-A deposition. Importantly, we identify CAL1 to be required for dCENP-C loading onto chromatin in cooperation with dCENP-A nucleosomes, thus closing the epigenetic loop to ensure dCENP-C and dCENP-A replenishment during the cell division cycle. Finally, we show that all three factors are sufficient for dCENP-A propagation and propose a model for the epigenetic inheritance of *Drosophila* centromere identity.

## Highlights

• The dCENP-A targeting domain is larger than in humans and matches CAL1 binding
• CAL1 loads dCENP-C onto chromatin in the presence of dCENP-A-nucleosomes
• Oligomerization of CAL1 and dCENP-C play crucial roles in centromere propagation
• dCENP-C, CAL1, and dCENP-A are sufficient for *Drosophila* centromere inheritance

## Graphical Abstract

**Figure:**
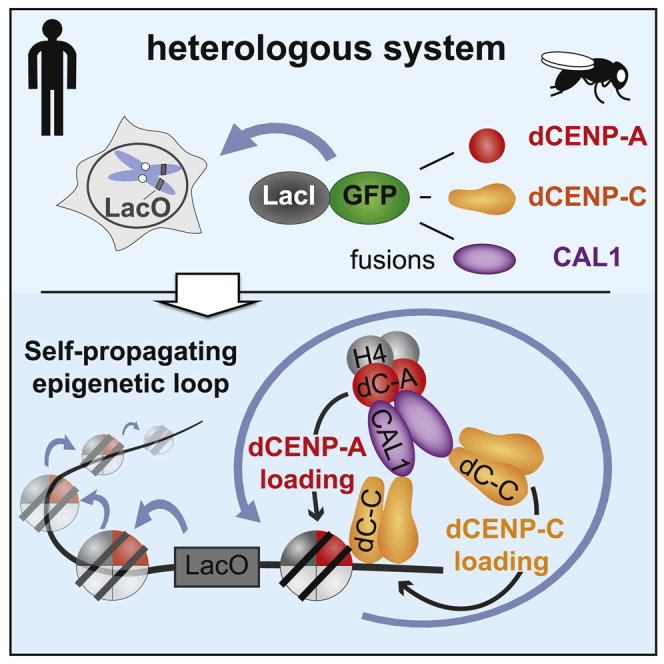

## Teaser

Centromeres are epigenetically identified by the histone-variant CENP-A. Using a heterologous system, Roure et al. investigate how *Drosophila* centromeres are maintained through cell generations and show that the chaperone CAL1 acts as a dynamic loading factor for dCENP-A and dCENP-C to sustain a self-propagating loop of centromere inheritance.

## Introduction

Centromeres are essential chromosomal regions that ensure the faithful distribution of genetic information during cell division. Most eukaryotic centromeres are defined by the presence of specialized nucleosomes containing the centromere-specific histone H3 variant, called centromere protein (CENP)-A (Black and Cleveland, 2011), and not by DNA sequence. Consistent with the epigenetic nature of centromeres, CENP-A is found in every identified neocentromere (Marshall et al., 2008), and ectopic targeting of CENP-A to a non-centromeric chromosomal locus is sufficient to generate a functional and heritable centromere (Barnhart et al., 2011, Logsdon et al., 2015, Mendiburo et al., 2011). The key function of CENP-A is to provide a self-propagating platform that directs kinetochore assembly, a macromolecular structure that mediates chromosome attachment to spindle microtubules during mitosis and meiosis.

To ensure epigenetic inheritance, the centromere mark must stably persist through the multiple division cycles of a cell. Unlike the canonical histone H3, CENP-A nucleosomes are not replenished during DNA replication but from late mitosis through Gap 1 (G1) phase (Dunleavy et al., 2012, Jansen et al., 2007, Lidsky et al., 2013, Schuh et al., 2007). One attractive model for the self-propagation of CENP-A is an epigenetic loop, where one or more adaptors recognize and direct the deposition of new CENP-A. In humans, CENP-A (hCENP-A) deposition requires the coordinated activity of several factors: the hCENP-A-specific histone chaperone HJURP, hCENP-C, and the Mis18 complex (McKinley and Cheeseman, 2016). The members of the Mis18 complex have been shown to interact with hCENP-C (Dambacher et al., 2012, Moree et al., 2011) and to recruit HJURP to centromeres (Barnhart et al., 2011, Wang et al., 2014) to promote loading of hCENP-A. Direct binding of hCENP-C to hCENP-A nucleosomes (Carroll et al., 2010, Kato et al., 2013) closes the loop, thereby providing a mechanism to ensure CENP-A propagation in human centromeres.

In *Drosophila melanogaster*, in addition to CENP-A (dCENP-A, also known as CID or cenH3), only two centromere proteins have been identified so far: the dCENP-A-specific chaperone CAL1, which mediates dCENP-A deposition (Chen et al., 2014), and *Drosophila* CENP-C (dCENP-C) (Heeger et al., 2005). CAL1, dCENP-C, and dCENP-A have been shown to be interdependent for centromere localization and function (Erhardt et al., 2008, Schittenhelm et al., 2010). However, in contrast to their human counterparts, dCENP-C and dCENP-A appear to interact only indirectly via the bridging factor CAL1, which binds dCENP-A through its N-terminal domain and dCENP-C through its C-terminal domain (Schittenhelm et al., 2010). CAL1 has been shown to be sufficient for dCENP-A nucleosome assembly and it has been proposed that dCENP-C mediates CAL1/dCENP-A recruitment to centromeres (Chen et al., 2014). However, how dCENP-C associates with the centromere and how centromeric chromatin is epigenetically propagated are not understood.

Although analysis of dCENP-A, dCENP-C, and CAL1 in their “natural” environment in *Drosophila* cells has provided insights into their roles in maintaining centromere identity, all three factors exhibit dependencies on each other for function and protein stability. The use of a heterologous system where none of the three proteins are essential for viability is unaffected by these complexities. Hence, to explore this possibility, we took advantage of the pronounced evolutionary divergence between the *Drosophila* and human centromere components. Using the LacI/LacO system, we artificially targeted the three *Drosophila* centromere proteins dCENP-A, dCENP-C, and CAL1 to chromosomally integrated LacO arrays in human U2OS cells to dissect their interactions and role in dCENP-A inheritance in unprecedented detail. First, we generated histone H3/dCENP-A chimeras to identify the *Drosophila* CENP-A centromere targeting domain as well as the interaction domain of dCENP-A with CAL1. LacI/LacO targeting further revealed the joint roles of both CAL1 and dCENP-A in recruiting dCENP-C to chromatin and highlighted the importance of dCENP-C and CAL1 self-association for their interactions and dCENP-A deposition. Finally, we showed that these three factors are sufficient for propagation of dCENP-A and proposed a model for the epigenetic inheritance of centromere identity in *Drosophila*.

## Results

### Identification of the Centromere Targeting Domain of *Drosophila* CENP-A

**Figure 1:**
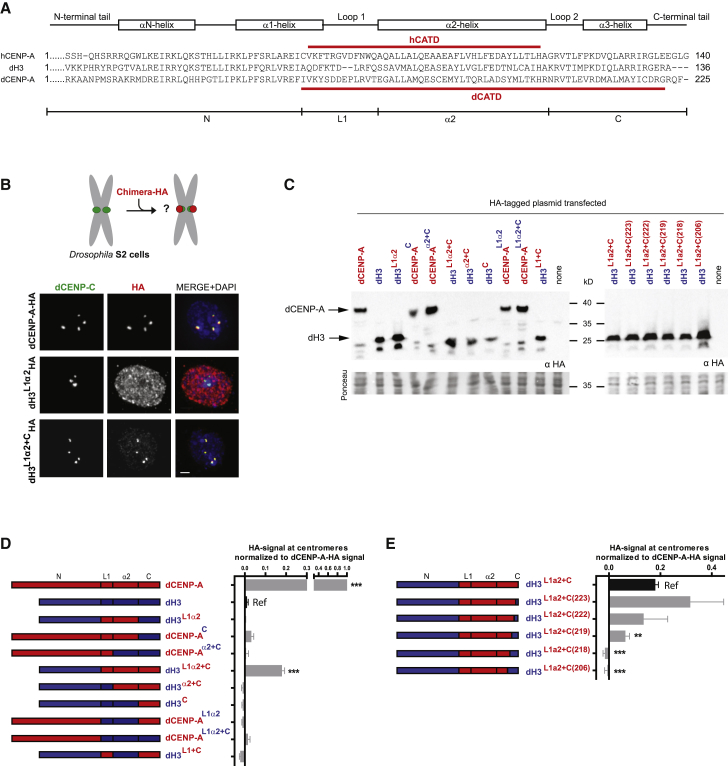
The CATD of CENP-A in *Drosophila* Is Larger than in Humans and Includes the α3 Helix (A) *Drosophila* CENP-A was divided into four domains: the N-terminal N from residues 1 to 160 (corresponding to residues 1 to 75 in dH3); the L1 domain from residues 161 to 173 contains loop L1 (residues 76 to 86 in dH3); the α2 domain, which contains helix α2 (residues 174 to 202 in dCENP-A and residues 87 to 115 in dH3); and the C-terminal C from residues 203 to 225 (residues 116 to 136 in dH3). (B) Experimental scheme and representative IF images of HA-tagged WT dCENP-A, dH3^L1α2^, and dH3^L1α2+C^ chimera expression patterns in S2 *Drosophila* cells. dCENP-C marks *Drosophila* centromeres. (C) Western blot analysis of expression levels of HA-tagged dCENP-A/dH3 chimeras using α-HA antibody. (D and E) Quantitation of indicated HA-tagged dCENP-A/dH3 chimera mean intensities at centromeres normalized to HA-tagged dCENP-A mean intensity at centromeres. Scale bar, 1μm. Error bars show SEM. Asterisks denote significant differences (^∗∗^p < 0.01; ^∗∗∗^p < 0.001); absence of an asterisk denotes a non-significant difference. The reference sample for statistical analysis is indicated as Ref.

To determine the region of *Drosophila* CENP-A required for its localization to centromeres, we designed a collection of chimeric dCENP-A/dH3 variants in which one or several domains of the histone dH3 were replaced by the corresponding domains of histone dCENP-A. The secondary structure of the histone fold is composed of three helices (α1, α2, and α3), which are connected by two loops (L1 and L2) (Figure 1A). Despite the divergence in amino-acid composition (overall ≈20%, histone fold ≈38% identity), dCENP-A mainly differs from dH3 in the longer loop 1 and N-terminal tail (Figure 1A). In human cells L1 and the α2 helix of hCENP-A are sufficient to target an H3 chimera to centromeres and are hence named the CENP-A-targeting domain (hCATD; Figure 1A) (Black et al., 2004). We divided *Drosophila* CENP-A and H3 into four regions—an N-terminal part (N), the L1 loop, helix α2, and a C-terminal part (C)—and expressed variants of dCENP-A/dH3 chimera fused to the hemagglutinin (HA) tag in *Drosophila* Schneider S2 cells (Figures 1A–1D).

Targeting of each chimera to endogenous *Drosophila* centromeres was assessed by quantitative immunofluorescence (IF) where the intensity of signal was measured (Figures 1B and 1D). Western blotting (WB) was performed to confirm that signal intensity at centromeres did not reflect protein expression levels (Figure 1C). We observed that, unlike in humans, the L1 and α2 regions of dCENP-A (dH3^**L1α2**^) are not sufficient, in line with Moreno-Moreno et al. (2011) and Vermaak et al. (2002), and that inclusion of the C region is required for dCENP-A/dH3 chimera to display centromere localization (dH3^**L1α2+C**^; Figures 1B and 1D). The L1 and α2 regions are both necessary, given that omittance of any of these two regions (dH3^α2+C^ and dH3^L1+ C^) interfered with centromere targeting (Figure 1D). Although the N region of dCENP-A is neither necessary nor sufficient for centromere targeting, it might still contribute to targeting, as the dH3^**L1α2+C**^ chimera was less efficient than wild-type dCENP-A (around 20%; Figure 1D). Narrowing down further the domain required for centromere targeting showed that the α3 helix in the C-terminal region was absolutely essential and only the last three residues of dCENP-A can be omitted for centromere localization (Figure 1E). Taken together, the *Drosophila* centromere targeting domain comprises residues 161-222, extending the human CATD up to the α3 helix, and is therefore significantly larger in dCENP-A (Figure 1A).

### Development of a Heterologous System to Reconstitute dCENP-A Loading

The CATD of human CENP-A is also the domain bound by its chaperone HJURP (Bassett et al., 2012, Foltz et al., 2009, Hu et al., 2011). Having defined the dCATD, we next sought to determine the domain of interaction between dCENP-A and its chaperone CAL1. To prevent interference due to the presence of endogenous centromere proteins in *Drosophila* cells, we developed a heterologous system in which *Drosophila* proteins were expressed in human cells.

**Figure 2:**
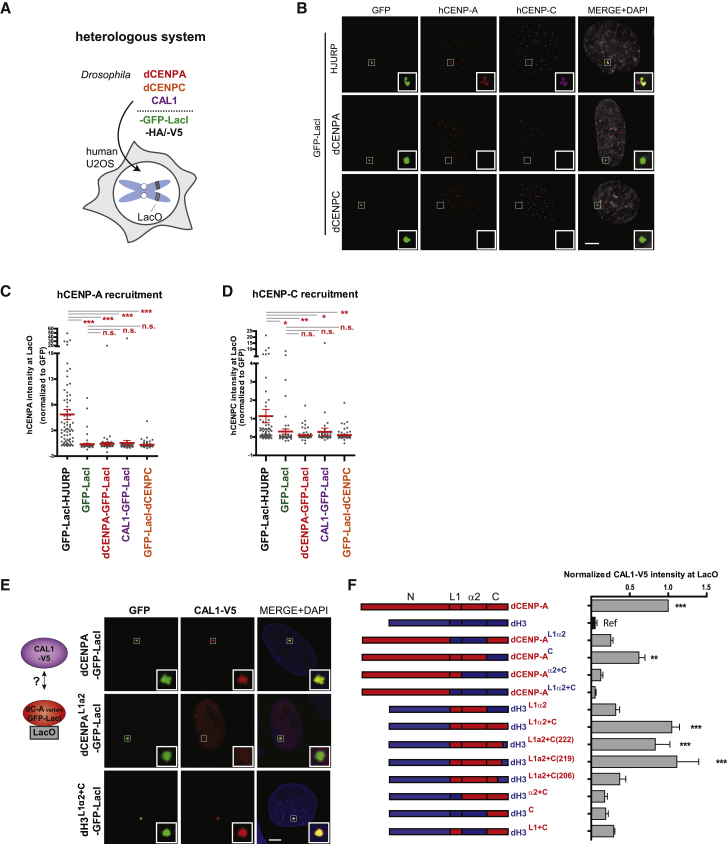
Human and *Drosophila* Centromere Factors Required for Establishing Centromere Identity Do Not Interfere in the Heterologous System (A) Cartoon illustrating the experimental setup of the heterologous system expressing *Drosophila* centromere proteins in human cells. (B) Representative IF images used to quantify hCENP-A and hCENP-C recruitment to LacO arrays by HJURP (pos. control) and *Drosophila* centromere factors fused to GFP-LacI in U2OS cells. (C and D) Quantitation of normalized hCENP-A (C) and hCENP-C (D) mean intensities at LacO upon tethering the indicated proteins fused to GFP-LacI. Insets show magnification of the boxed regions. Scale bar, 5 μm. Error bars show SEM (^∗^p < 0.05; ^∗∗^p < 0.01; ^∗∗∗^p < 0.001; n.s., not significant). (E) Experimental scheme and representative IF images of CAL1-V5 recruitment to the LacO arrays by GFP-LacI-tagged dCENP-A, dCENP-A^L1α2^, and dH3^L1α2+C^ in U2OS cells. (F) Quantitation of CAL1-V5 mean intensity at LacO upon tethering of the indicated GFP-LacI-tagged chimeras, normalized to its mean intensity at LacO upon tethering of GFP-LacI-tagged dCENP-A. (See also Figure S1.) Error bars show SEM. Asterisks denote significant differences (^∗∗^p < 0.01; ^∗∗∗^p < 0.001); absence of an asterisk denotes a non-significant difference. Insets show magnification of the boxed regions. Scale bar, 5 μm. The reference sample for statistical analysis is indicated as Ref.

Both experimental and bioinformatic approaches show little conservation between human and *Drosophila* for factors involved in CENP-A replenishment, revealing a low degree of sequence identity for some components in *Drosophila* (CENP-A, 21.2%; CENP-C, 11.4%) and the complete absence of others (HJURP, Mis18 complex; Figure S1G) (McKinley and Cheeseman, 2016, Westermann and Schleiffer, 2013). Thus, we reasoned that human centromere proteins were unlikely to interact or interfere with exogenously expressed *Drosophila* centromere proteins, yet other cellular components would help by providing a similar chromatin context and physiological conditions to support protein interactions and function. Here, we utilized U2OS cells containing integrated mixed LacO and TetO arrays and transiently expressed *Drosophila* centromere proteins fused to the *lac* repressor (LacI) (Figure 2A) (Barnhart et al., 2011, Janicki et al., 2004). The LacI-fusion proteins can be specifically targeted to the LacO arrays and analyzed for the co-recruitment of other ectopically expressed *Drosophila* proteins of interest (Zolghadr et al., 2008).

To confirm that there is no crosstalk between the human and the *Drosophila* centromere proteins in these cells, we expressed the *Drosophila* centromere proteins CAL1, dCENP-A, and dCENP-C, as well as the human centromere protein HJURP as positive control, fused to a tandem GFP-LacI tag. We first checked if these GFP-LacI-tagged proteins were properly expressed in U2OS cells by WB (Figure S1A). Although CAL1 and dCENP-C could not be detected by this approach, probably due to protein instability, IF experiments revealed that these factors were similarly expressed and properly targeted to LacO arrays in U2OS cells (Figures 2B, S1B, and S1C).

Each GFP-LacI fusion was then tested by IF for their ability to recruit the human centromere proteins (hCENP-A and hCENP-C) to the LacO arrays in U2OS cells (Figures 1B–1D and S1C). As expected, the human centromere protein HJURP significantly recruited hCENP-A (Figures 2B and 2C) and hCENP-C (Figures 2B and 2D) to the LacO arrays, yet they were not detected by tethering *Drosophila* CENP-A, CENP-C, or CAL1, similar to the GFP-LacI negative control (Figures 2B–2D and S1C). This was further supported by chromatin immunoprecipitation (ChIP) experiments using HA-tagged hCENP-A, hCENP-C, and HJURP. We confirmed the presence of all three human factors on endogenous centromeric alpha-satellite DNA, yet no signal could be detected on LacO DNA in the presence of the targeted *Drosophila* centromere proteins CAL1, dCENP-A, and dCENP-C (Figures S1D–S1F). While we noticed that transiently transfected dCENP-C was occasionally localized close to human centromeres, high-resolution imaging revealed that dCENP-C did not overlap with endogenous human centromeres (identified by hCENP-A). Instead, it was recruited to pericentric regions for currently unknown reasons (Figure S1H). We therefore decided to exclusively focus on the LacO array, where our analysis demonstrated the complete absence of direct interaction or proximal localization. Moreover, the absence of interactions between human and *Drosophila* centromere proteins was confirmed in a reciprocal heterologous system where human centromere proteins were expressed in *Drosophila* S2 cells containing a LacO plasmid (Logsdon et al., 2015). We conclude there is no interaction of endogenous human centromere proteins with the exogenously expressed *Drosophila* centromere proteins in our heterologous system.

### The dCATD Is Required for Wild-Type Levels of CAL1/dCENP-A Interaction

To determine the domain of interaction of dCENP-A with CAL1, dCENP-A/dH3 chimeras expressed as GFP-LacI fusions were tethered to LacO arrays in U2OS transiently expressing V5-tagged CAL1 to analyze its localization (Figures 2E and S1I). Although weak association between CAL1 and the dH3^**L1α2**^ chimera was detected, the presence of the N (dCENP-A^**C**^) or C parts of dCENP-A (dH3^**L1α2+C**^, dH3^**L1α2+C(222)**^, dH3^**L1α2+C(219)**^, and dH3^**L1α2+C(206)**^) significantly increased the co-recruitment of CAL1 to LacO arrays (Figure 2F). Generally, the level of interaction between the chimeras and CAL1 correlated with their ability to be targeted to *Drosophila* centromeres, with the full dCATD containing chimeras displaying the strongest recruitment of CAL1-V5 similar to wild-type dCENP-A (Figures 1D and 1E). As an exception, the chimera dCENP-A^**C**^ exhibited lower but significant levels of CAL1 recruitment (60% compare to wild-type [WT] dCENP-A; Figure 2F), yet hardly localized to centromeres (Figure 1D). This observation suggests that, although multiple regions across dCENP-A including its N region contribute to CAL1 binding, only the full dCATD provides the high level of association required for centromere targeting. In summary, these results show that the domain of interaction of dCENP-A with CAL1 is similar to the dCENP-A centromere targeting domain.

### dCENP-C Can Recruit the CAL1/dCENP-A/H4 Complex by Directly Interacting with CAL1

**Figure 3:**
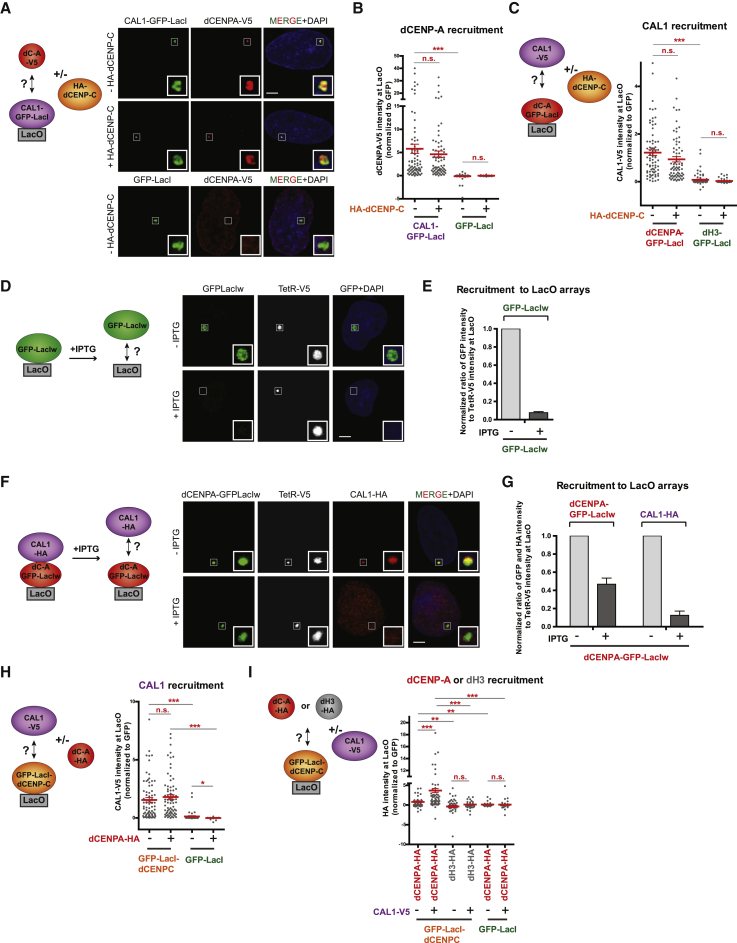
dCENP-C Recruits the CAL1/dCENP-A Complex to Chromatin (A) Experimental scheme and representative IF images of dCENP-A-V5 recruitment to the LacO arrays by CAL1-GFP-LacI or GFP-LacI, +/− HA-dCENP-C, in U2OS cells. (B) Quantitation of normalized dCENP-A-V5 mean intensity at LacO upon tethering of CAL1-GFP-LacI or the control GFP-LacI, +/− HA-dCENP-C (for % cells see Figure S2A). (C) Experimental scheme and quantitation of normalized CAL1-V5 mean intensity at LacO upon tethering of GFP-LacI-tagged dCENP-A or dH3, +/− HA-dCENP-C (for % cells see Figure S2C). (D) Experimental scheme and representative IF images of GFP-LacIw recruitment to the LacO arrays, +/− IPTG treatment in U2OS cells. (E) Quantitation of normalized GFP-LacIw mean intensity at LacO, +/− IPTG treatment. (F) Experimental scheme and representative IF images of CAL1-HA recruitment to the LacO arrays by dCENP-A-GFP-LacIw, +/− IPTG treatment in U2OS cells. (G) Quantitation of normalized dCENP-A-GFP-LacIw and CAL1-HA mean intensities at LacO, +/− IPTG treatment. (H) Experimental scheme and quantitation of normalized CAL1-V5 mean intensity at LacO upon tethering of GFP-Lac-dCENP-C or GFP-LacI, +/− dCENP-A-HA (for % cells see Figure S2H). (I) Experimental scheme and quantitation of normalized HA-tagged dCENP-A or dH3 mean intensities at LacO upon tethering of GFP-Lac-dCENP-C or GFP-LacI, +/− CAL1-V5 (for % cells see Figure S2J; see also Figure S2). Scale bar, 5 μm. Insets show magnification of the boxed regions. Error bars show SEM (^∗^p < 0.05; ^∗∗^p < 0.01; ^∗∗∗^p < 0.001; n.s., not significant).

Taking advantage of our heterologous system to investigate direct interactions, we decided to further dissect step-by-step dCENP-A and CAL1 recruitment to chromatin. We tested whether the previously reported direct interaction of dCENP-A and CAL1 could be reproduced (Chen et al., 2014, Schittenhelm et al., 2010), and potentially be enhanced by the presence of dCENP-C. As expected, GFP-LacI-tethered CAL1 efficiently recruited dCENP-A to the LacO (Figures 3A and 3B; 97% cells; Figure S2A) and vice versa (Figures 3C and S2B; 100% cells; Figure S2C), but was unaffected by the presence or absence of HA-dCENP-C (Figures 3A–3C and S2A–S2C), confirming previous findings that CAL1 and dCENP-A directly interact.

To investigate if dCENP-A nucleosomes maintain affinity for CAL1, we first checked whether dCENP-A-GFP-LacI is incorporated into chromatin assembled on LacO arrays. As previously shown with human CENP-A (Barnhart et al., 2011, Logsdon et al., 2015), we expected to find two pools of LacI-tagged dCENP-A at the LacO arrays: one bound to LacO through the LacI tether and sensitive to the LacI allosteric effector molecule IPTG and another that is stably assembled into nucleosomes and therefore IPTG insensitive. dCENP-A was tagged with a mutated LacI variant (LacIw) with increased IPTG sensitivity (Loiodice et al., 2014) and co-transfected with HA-tagged CAL1 and V5-tagged Tet repressor (TetR) to independently mark the arrays. 48 h after transfection, cells were treated with IPTG and analyzed to determine the IPTG sensitivity of dCENP-A and CAL1. While we found that the control GFP-LacIw is almost entirely displaced (Figures 3D and 3E), roughly half of the pool of dCENP-A-GFP-LacIw was retained at the LacO arrays after IPTG treatment (Figures 3F and 3G), suggesting that a fraction of dCENP-A had assembled into chromatin. As a control for proper dCENP-A incorporation into human chromatin, we mutated two hydrophobic residues in dCENP-A, Y187A, and L188Q predicted to disrupt its interaction with histone H4 (equivalent to F101 and L102 in the hCENP-A crystal structure; Sekulic et al., 2010) and prevent its chromatin loading. Indeed, this dCENP-A mutant was completely removed from chromatin upon addition of IPTG (Figure S2D), suggesting that WT dCENP-A can form a nucleosome with human H4 and be properly incorporated into chromatin. Gel filtration and size exclusion chromatography combined multi-angle light scattering (SEC-MALS) experiments confirmed that CAL1 forms a complex with recombinant dCENP-A and human histone H4 (Figures S2E and S2F). Importantly, the co-recruitment of CAL1-HA to the LacO arrays by dCENP-A-GFP-LacIw was completely IPTG sensitive (Figures 3F and 3G), indicating that CAL1 could not bind nucleosomal dCENP-A and that only the pool of non-nucleosomal dCENP-A was available for binding its chaperone CAL1, presumably mimicking the soluble form of the protein. Together, these results strongly suggest that once recruited to centromeres and after chromatin loading of dCENP-A, CAL1 behaves similarly to human HJURP and dissociates from nucleosomal dCENP-A (Barnhart et al., 2011, Logsdon et al., 2015). Importantly, it also shows that nucleosomal dCENP-A does not directly recruit a new CAL1/dCENP-A/H4 soluble complex.

We next investigated whether dCENP-C was able to directly recruit CAL1, dCENP-A, or both to the chromatin. We observed that, when GFP-LacI-dCENP-C was tethered to the LacO, it could recruit CAL1-V5 very efficiently unaffected by presence or absence of dCENP-A-HA (Figures 3H and S2G; 97% and 94% cells, respectively; Figure S2H), compared to the GFP-LacI negative control (Figure 3H; 7% and 4% cells; Figure S2H). Surprisingly, we observed low but significant direct interactions between GFP-LacI-dCENP-C and dCENP-A-HA in the absence of CAL1 (Figures 3I and S2I; 43% of cells with dCENP-A localization versus 24% with dH3; Figure S2J), similar to direct interactions described between hCENP-C and hCENP-A (Carroll et al., 2010). However, the recruitment of dCENP-A was strongly enhanced by CAL1-V5 (Figures 3I and S2I; 73% of cells with dCENP-A localization versus 5% with dH3; Figure S2J). Hence, this setup mimics the first step in dCENP-A loading and suggests that recognition of dCENP-C by CAL1 is required to recruit the CAL1/dCENP-A/dH4 soluble complex for the deposition of dCENP-A.

### CAL1 Self-Association Is Not Required for Binding but for Deposition of dCENP-A

**Figure 4:**
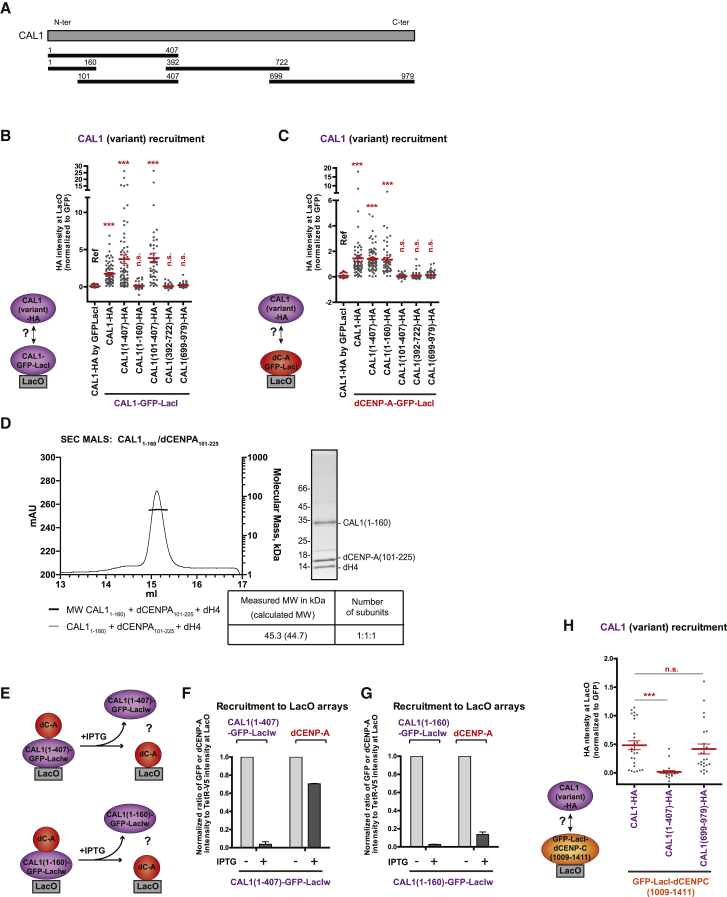
Self-Association of CAL1 Is Not Required for Its Interaction with dCENP-A and dCENP-C but Is Essential for Loading dCENP-A into Chromatin (A) The CAL1 N-terminal, middle, and C-terminal regions used in this study are represented by black horizontal lines with numbers indicating amino-acid positions. (B and C) Experimental schemes and quantitation of normalized HA-tagged CAL1 and CAL1 fragment mean intensities at LacO upon tethering of CAL1-GFP-LacI (B), dCENP-A-GFP-LacI (C), or GFP-LacI (B and C, first lane). (D) SEC-MALS experiments performed with recombinant CAL1(1-160) in complex with dCENP-A(101-225) and dH4. Elution volume (ml, x axis) is plotted against absorption at 280 nm (mAu, left y axis) and molecular mass (kDa, right y axis). Tables show measure molecular weight (MW) and calculated MW. Mw/Mn = 1.001. (E) Experimental scheme for the experiments shown in (F) and (G). (F and G) Quantitation of normalized dCENP-A, CAL1(1-407)-GFP-LacIw (F), and CAL1(1-160)-GFP-LacIw (G) mean intensities at LacO, +/− IPTG treatment. (H) Quantitation of normalized HA-tagged CAL1 and CAL1 fragment mean intensities at LacO upon tethering of GFP-LacI-CENP-C(1009-1411). Error bars show SEM (^∗∗∗^p < 0.001; n.s., not significant). The reference sample for statistical analysis is indicated as Ref. See also Figures S3 and S4.

Dimerization of HJURP has been shown to be essential for the deposition of hCENP-A nucleosomes (Zasadzińska et al., 2013). We first checked if the property of self-association was conserved in the *Drosophila* histone chaperone CAL1. We observed that CAL1-GFP-LacI was able to recruit CAL1-HA to the arrays, suggesting that CAL1 could self-associate (Figure 4B, first and second lanes; Figure S3A). To determine the CAL1 self-association domain, we generated different CAL1 fragments corresponding to the N-terminal (1–407), middle part (392–722), and C-terminal (699–979) of CAL1 as previously described (Schittenhelm et al., 2010) (Figure 4A) and performed the above-described LacI/LacO recruitment assay. We found that only the fragment (1–407) was recruited by CAL1-GFP-LacI to the LacO arrays (Figures 4B and S3A), suggesting its ability to self-associate. By generating two fragments from residues 1 to 160 and from 101 to 407 (Chen et al., 2014) (Figure 4A), the CAL1 self-association domain was further narrowed down to the region between residues 161 and 407 of the N-terminal part (Figures 4B and S3A). SEC-MALS analysis further confirmed that the CAL1(1–160) fragment behaves as a monomer (Figure S3B).

As CAL1(1–407) was previously shown to interact with dCENP-A (Schittenhelm et al., 2010), we asked if CAL1 self-association was required for its interaction with dCENP-A. CENP-A-GFP-LacI was tethered to the LacO arrays to determine the recruitment of the different CAL1-HA fragments. We first confirmed that the interaction domain of CAL1 with dCENP-A is contained between residues 1 and 160 of its N-terminal part (Figures 4C and S3C). This is consistent with previous *in vitro* experiments using the same fragment (Chen et al., 2014) and was further confirmed by SEC-MALS experiments (Figure 4D). Together, these results demonstrate that different regions of CAL1 are responsible for its self-association and interaction with dCENP-A, suggesting that they are functionally separate.

To investigate further the role of CAL1 self-association in dCENP-A deposition, we next sought to understand the stoichiometry of the CAL1/dCENP-A complex by performing SEC-MALS experiments in the presence of CAL1(1–160), dCENP-A (101–255; lacking the N-terminal tail), and dH4. Interestingly, CAL1(1–160) bound only one (dCENP-A/dH4) dimer *in vitro* (Figure 4D), suggesting that the self-association domain could be necessary to bring together two (dCENP-A/dH4) dimers to assemble a tetramer on the chromatin. To test this hypothesis, we determined whether CAL1 self-association was required for dCENP-A assembly into chromatin *in vivo* by analyzing the persistence of dCENP-A at the LacO arrays after IPTG treatment upon tethering of GFP-LacIw-fusion of CAL1(1–407) or CAL1(1–160) (Figure 4E). As expected, most of the dCENP-A recruited by CAL1(1–407)-GFP-LacIw remained on the arrays after IPTG treatment (Figures 4F and S3D), showing that dCENP-A was properly incorporated into chromatin by CAL1(1–407). Importantly, dCENP-A recruited by CAL1(1–160)-GFP-LacIw was IPTG sensitive (Figures 4G and S3E), demonstrating that CAL1(1–160) missing its self-association domain was not able to incorporate dCENP-A into chromatin. This is in contrast to *in vitro* experiments, demonstrating that CAL1(1–160) is sufficient to assemble CENP-A nucleosomes using a plasmid supercoiling assay (Chen et al., 2014). In summary, our results strongly suggest that CAL1 self-association is necessary for deposition of the tetramer dCENP-A/dH4 to the chromatin. Thus, CAL1 behaves similar to its human counterpart HJURP despite being evolutionary unrelated.

### CAL1 Self-Association Is Not Required for Interaction with dCENP-C

We next considered that CAL1 self-association might also be important for its interaction with dCENP-C. It has previously been shown that CAL1 and dCENP-C interact through their C-terminal domains (Schittenhelm et al., 2010). The C-terminal fragment of dCENP-C (residues 1009–411) tethered to LacO arrays was analyzed for its ability to recruit full-length CAL1, CAL1(699–979) (no self-association), or the negative control CAL1(1–407) (self-associating, but not interacting with dCENP-C). We observed that dCENP-C(1009–1411) could interact with full-length CAL1 and CAL1(699–979) with similar efficiencies (Figures 4H and S4A), suggesting that CAL1 self-association is not required for its interaction with dCENP-C.

We also noticed that full-length dCENP-C recruited full-length CAL1 more effectively than its C-terminal fragment CAL1(699–979) (Figure S4B). This suggests that the N terminus of CAL1 could contribute to optimal CAL1-dCENP-C binding due to its ability to self-associate. In agreement, expressing the N- (1–407) or C-terminal (699–979) regions of CAL1 in S2 cells reveals that neither was sufficient to localize to *Drosophila* centromeres (Figures S4C and S4D), consistent with CAL1 centromere localization depending on both its N- and C-terminal regions and the presence of dCENP-A and dCENP-C (Erhardt et al., 2008, Goshima et al., 2007, Schittenhelm et al., 2010). In summary, these data suggest that although self-association of CAL1 subunits is not required for dCENP-C binding, CAL1 is likely recruited by dCENP-C as a multimer, possibly a dimer in complex with two dCENP-A/dH4.

### CAL1 Is an Efficient Loader of dCENP-C in the Presence of Nucleosomal dCENP-A

**Figure 5:**
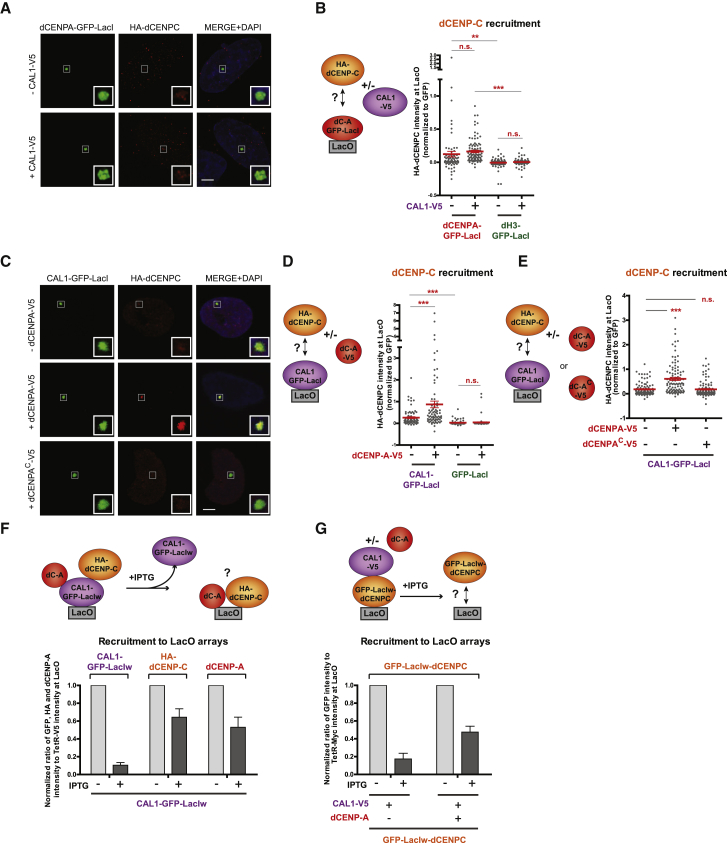
dCENP-C Is Loaded by CAL1 to LacO Arrays Containing dCENP-A Nucleosomes (A) Representative IF images of HA-dCENP-C recruitment to the LacO arrays by dCENP-A-GFP-LacI, +/− CAL1-V5, in U2OS cells. (B) Experimental scheme and quantitation of normalized HA-dCENP-C mean intensity at LacO upon tethering of GFP-LacI-tagged dCENP-A- or dH3, +/− CAL1-V5 (for % cells see Figure S5A). (C) Representative IF images of HA-dCENP-C recruitment to the LacO arrays by CAL1-GFP-LacI, +/− dCENP-A-V5 or dCENP-A^C^-V5, in U2OS cells. (D) Experimental scheme and quantitation of normalized HA-dCENP-C mean intensity at LacO upon tethering of CAL1-GFP-LacI or GFP-LacI, +/− dCENP-A-V5 (for % cells see Figure S5B). (E) Experimental scheme and quantitation of normalized HA-dCENP-C mean intensity at LacO upon tethering of CAL1-GFP-LacI, +/− V5-tagged dCENP-A or dCENP-A^C^. (F) Experimental scheme and quantitation of normalized CAL1-GFP-LacIw, HA-dCENP-C, and dCENP-A mean intensities at LacO, +/− IPTG treatment. (G) Experimental scheme and quantitation of normalized GFP-LacIw-dCENP-C mean intensity at LacO in the presence of CAL1-V5, +/− dCENP-A and +/− IPTG treatment. Scale bar, 5 μm. Insets show magnification of the boxed regions. Error bars show SEM (^∗∗^p < 0.01; ^∗∗∗^p < 0.001; n.s., not significant). See also Figure S5.

One aspect of the epigenetic self-propagation loop of dCENP-A that remains poorly understood is how dCENP-C is recruited to the centromeres. To address this question, we first analyzed the recruitment of dCENP-C by dCENP-A, in the presence or absence of CAL1. We found that tethered dCENP-A-GFP-LacI could recruit low levels of HA-dCENP-C in the absence of CAL1-V5 as compared to the H3-GFP-LacI control (Figures 5A and 5B; 17% cells versus 3%; Figure S5A), confirming the weak direct interaction of dCENP-A with dCENP-C observed before (Figure 3I). The presence of CAL1 only slightly increased dCENP-C recruitment by dCENP-A-GFP-LacI as the intensity of the HA-dCENP-C signal at the LacO was not significantly different with or without CAL1 (Figures 5A and 5B; 55% cells; Figure S5A), presumably because CAL1 and dCENP-C do not appear to form a soluble complex (Mellone et al., 2011). We conclude that dCENP-A is unlikely to act as the only dCENP-C recruitment factor.

We next determined the ability of CAL1 to recruit dCENP-C. Based on the reported interactions between dCENP-C and CAL1 in yeast two-hybrid assays (Schittenhelm et al., 2010) and our own data using the LacI tether on dCENP-C (Figures 3H, S2G, and S2H), we were surprised to find that CAL1-GFP-LacI alone recruited only low levels of HA-dCENP-C to the LacO (Figures 5C and 5D; 31% cells; Figure S5B). Interestingly, HA-dCENP-C localization to the LacO was significantly enhanced in the presence of dCENP-A-V5 (Figures 5C and 5D; 76% cells; Figure S5B), indicating that CAL1 and dCENP-A cooperate for dCENP-C recruitment. The GFP-LacI tag on the C terminus of CAL1 did not prevent its association with dCENP-C, as N-terminally GFP-LacI-tagged CAL1 presented identical levels of interaction with dCENP-C (Figure S5C). Focusing on the role of dCENP-A, we investigated if nucleosomal or soluble dCENP-A in complex with CAL1 was important for dCENP-C recruitment. We first took advantage of the dCENP-A^C^ chimera, which interacts with CAL1 (Figure 2F), but cannot be stably incorporated into the chromatin, as shown by IPTG treatment experiments (Figure S2D). The ability of tethered CAL1-GFP-LacI to recruit HA-dCENP-C alone was tested in the presence of dCENP-A-V5 or the chimera dCENP-A^C^-V5 (Figures 5C, 5E, and S5D). The presence of dCENP-A-V5, but not dCENP-A^C^-V5 increased significantly the association of HA-dCENP-C with the LacO arrays relative to HA-dCENP-C alone. This suggested that dCENP-C has affinity for dCENP-A nucleosomes, which might contribute to both the recruitment and stable chromatin binding of dCENP-C on chromatin. To test this hypothesis, we first determined whether HA-dCENP-C recruitment by CAL1-GFP-LacIw was IPTG sensitive in the presence of dCENP-A (Figures 5F and S5E). As expected, CAL1-GFP-LacIw was almost completely displaced from the LacO following IPTG addition, whereas more than 50% of the dCENP-A pool was retained. We found more than half of the HA-dCENP-C pool also remained at the LacO array, suggesting that dCENP-C has affinity for dCENP-A chromatin. This was unknown for *Drosophila*, but is similar to what has been described for their mammalian homologs (Guse et al., 2011, Kato et al., 2013).

Tethering of GFP-LacIw-dCENP-C in cells expressing CAL1-V5 in the presence or absence of dCENP-A revealed that dCENP-C is significantly retained after IPTG treatment only in the presence of dCENP-A (Figures 5G, S5F, and S5G). This indicates that assembled dCENP-A nucleosomes allow retention of GFP-LacIw-dCENP-C following IPTG addition and does not require the continuous presence of CAL1. In summary, these data suggest that CAL1 in collaboration with dCENP-A is required to initially recruit dCENP-C, but only nucleosomal dCENP-A contributes to stable binding of dCENP-C to chromatin.

### dCENP-C Dimerization Is Required for CAL1 Association

**Figure 6:**
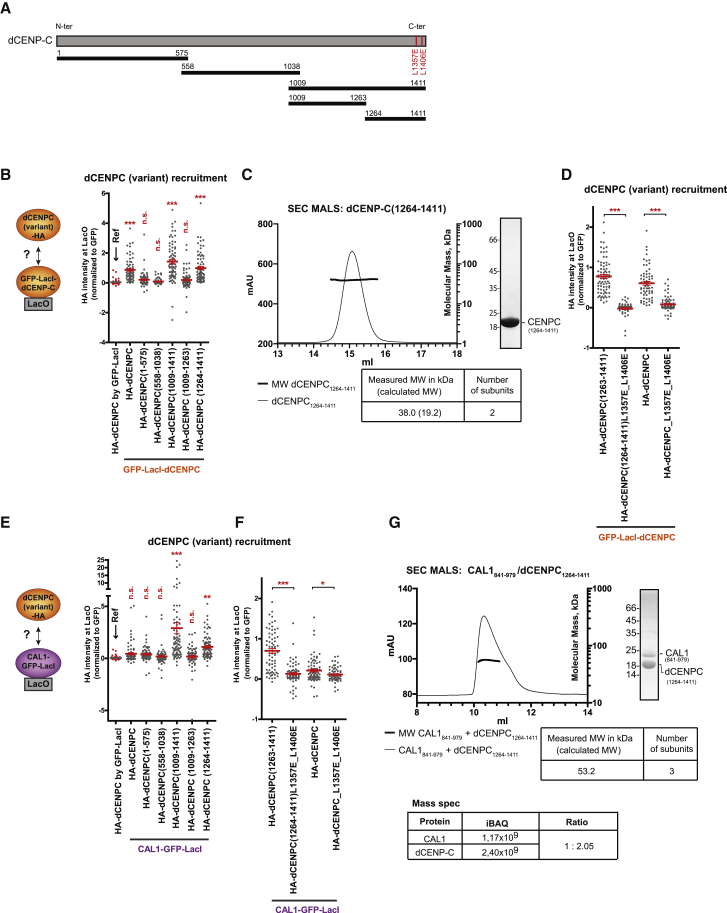
dCENP-C Dimerization Is Necessary to Interact with Monomeric CAL1 to Form a Trimer (A) The dCENP-C N-terminal, middle, and C-terminal regions used in this study are represented by black horizontal lines with numbers indicating amino-acid positions. (B) Experimental scheme and quantitation of normalized HA-tagged dCENP-C and dCENP-C fragment mean intensities at LacO upon tethering of GFP-LacI-dCENP-C or GFP-LacI (first lane). (C) SEC-MALS experiments performed with recombinant His-dCENP-C (1264-1411). Elution volume (ml, x axis) is plotted against absorption at 280 nm (mAu, left y axis) and molecular mass (kDa, right y axis). Tables show measure molecular weight (MW) and calculated MW. Mw/Mn = 1.001. (D) Quantitation of normalized HA-tagged wild-type and mutant dCENP-C fragment mean intensities at LacO upon tethering of GFP-LacI-dCENP-C. (E and F) Experimental scheme (E) and quantitation of normalized HA-tagged dCENP-C and dCENP-C fragment mean intensities at LacO upon tethering of CAL1-GFP-LacI (E and F) or GFP-LacI (E, first lane). (See also Figure S6.) Error bars show SEM (^∗^p < 0.05; ^∗∗^p < 0.01; ^∗∗∗^p < 0.001; n.s., not significant). (G) SEC-MALS experiments performed on the recombinant His-dCENP-C (1264-1411) and CAL1 (841-979) fragment complex. Bottom table shows the ratio of mass-spectroscopy-derived iBAQ peptide intensities of the fragments within the complex.

It has previously been shown that the C-terminal region of CENP-C is required for its dimerization in budding yeast (Mif2p) and humans (Cohen et al., 2008, Sugimoto et al., 1997). To determine if dimerization is conserved in *Drosophila* CENP-C, GFP-LacI-dCENP-C was tested for its ability to recruit transiently expressed full-length HA-dCENP-C. Significant HA-dCENP-C recruitment, compared to the GFP-LacI control, suggested that CENP-C in *Drosophila* was able to self-associate (Figure 6B, first and second lanes; Figure S6A). Different HA-tagged dCENP-C fragments corresponding to the N terminus (1–575), middle part (558–1038), and C terminus (1009–1411) of dCENP-C as previously published (Heeger et al., 2005) (Figure 6A) revealed that the C-terminal region of dCENP-C was sufficient for dCENP-C self-association (Figures 6B and S6A). Further dissection narrowed down the minimal self-association domain down to residues 1264–1411 (Figures 6A, 6B, and S6A) and SEC-MALS analysis of recombinant dCENP-C(1264–1411) showed dimer formation of this domain (Figure 6C).

The crystal structure of Mif2p revealed that three residues in the cupin-fold are required for Mif2p dimer formation (Cohen et al., 2008). Comparative structure analysis allowed prediction of a putative *Drosophila* CENP-C dimer and identified two leucine residues (positions 1357 and 1406) that could be required for dCENP-C dimer formation (Figures 6A and S6B). Mutations of these two residues (L1357E and L1406E) in the cupin fragment alone (1264–1411) or in full-length dCENP-C were sufficient to prevent its dimerization (Figures 6D and S6A), indicating that these two leucines are crucial for the formation of dCENP-C dimers.

To determine whether the dimerization domain of dCENP-C was required for association with CAL1, we tested the recruitment of the various dCENP-C fragments to the LacO arrays by CAL1-GFP-LacI (Figures 6E and S6C). Our analysis revealed that CAL1 associated with the dCENP-C(1264–1411) fragment, confirming previous observations using yeast two-hybrid analysis (Schittenhelm et al., 2010). Thus, the dCENP-C dimerization domain and the domain of interaction with CAL1 reside within the same region of dCENP-C. Mutations of the two leucine residues in the dimerization domain of dCENP-C also prevented its recruitment by LacO-tethered CAL1-GFP-LacI (Figures 6F and S6C), suggesting that dimerization of the cupin-fold of dCENP-C is necessary to associate with CAL1.

**Figure 7:**
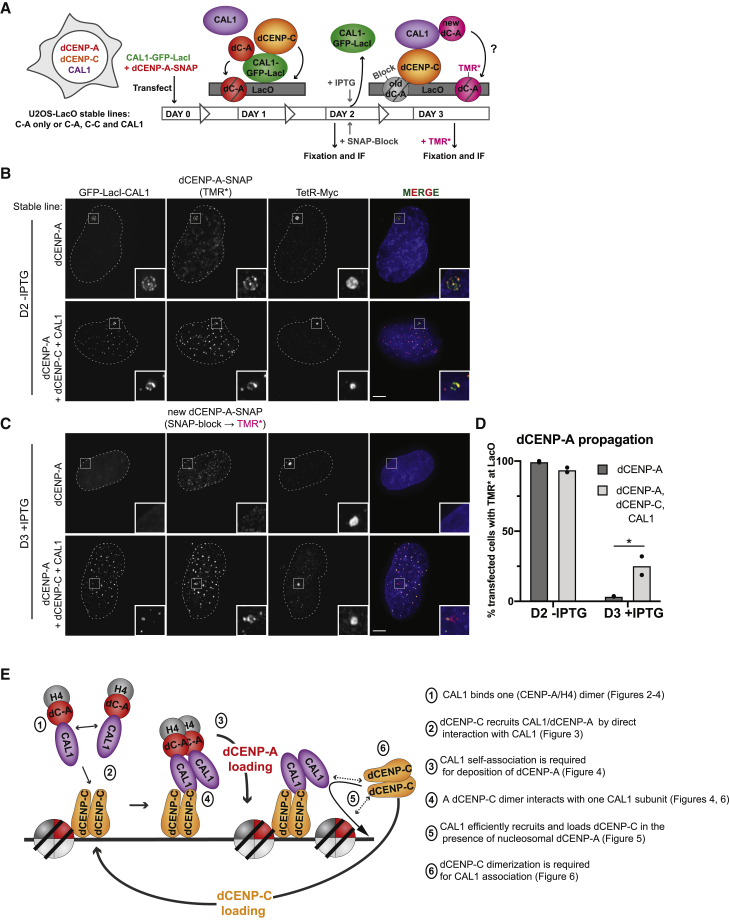
*Drosophila* dCENP-A Can Be Propagated at Ectopic Sites on Human Chromosomes (A) Experimental procedure to test epigenetic inheritance of dCENP-A in human cells. 48 h (day 2) after transfection of Cal1-GFP-LacI and dCENP-A-SNAP, cells were either stained with TMR^∗^ and subjected to IF or incubated with IPTG to remove CAL1-GFP-LacI binding and SNAP-Cell Block to quench existing SNAP-dCENP-A molecules. After further culturing for 24 h, cells were incubated with TMR-Star to stain newly synthesized SNAP-dCENP-A (day 3). (B and C) Representative IF images of the recruitment of dCENP-A to the LacO arrays 2 days (B) or 3 days (C) after transfection of CAL1-GFP-LacIw and dCENP-A-SNAP in U2OS-LacO stable cell line expressing all three *Drosophila* centromeric proteins or only dCENP-A. Insets show magnification of the boxed regions. Scale bar, 5 μm. (D) Quantification of the percentage of cells with TMR^∗^ signal at the LacO using imaging data of (B) and (C). Two experiments (dark circles) and their average (column) are shown. ^∗^p < 0.05. (See also Figure S7). (E) Model for centromere epigenetic inheritance in *Drosophila*. For dCENP-A (C-A in red) loading, a dCENP-C dimer (in orange) recruits a CAL1 dimer (in purple) in complex with 2× (dCENP-A/H4). For dCENP-C recruitment, one subunit of the CAL1 dimer is available for recruiting a soluble dCENP-C dimer, which is stabilized at the chromatin by dCENP-A nucleosomes.

Although we have demonstrated that CAL1 self-association is not required for its interaction with dCENP-C (Figure 4H), this does not rule out that two CAL1 monomers could bind the dCENP-C dimer in a 2:2 ratio. To reveal the stoichiometry, we performed SEC-MALS of a complex containing the C-terminal fragments of dCENP-C(1264–1411) and CAL1(841–979). Importantly, the molecular weight measurement indicated trimer formation, suggesting a 1:2 ratio for the CAL1:dCENP-C fragment complex (Figure 6G). This is further supported by the ratio of mass-spectroscopy derived-iBAQ (intensity-based absolute quantification) peptide intensities of the fragments within the complex, which is almost exactly 1:2 (Figure 6G) (Schwanhäusser et al., 2011). In summary, this shows that a dCENP-C dimer is bound by only one CAL1 monomer fragment. This finding has important implications for our understanding of how dCENP-C loading might be accomplished, as it leaves a second CAL1 subunit free to recruit an additional dCENP-C dimer (see Figure 7E and the Discussion section).

### CAL1, dCENP-C, and dCENP-A Are Sufficient for dCENP-A Propagation in Human Cells

To examine if the three *Drosophila* centromere proteins are sufficient to stably propagate dCENP-A in a heterologous system across continuous cell division cycles, we generated two U2OS-LacO-TetO lines: one stably expressing all three transgenes CAL1-V5, HA-dCENP-C, and dCENP-A (Figure S7B) and a control line only expressing dCENP-A (Figure S7B). Interestingly, similar to our previous observation of transiently transfected dCENP-C (Figure S1H), we noticed that in the triple transgene line, all three *Drosophila* proteins co-localized at pericentric regions of endogenous human centromeres. Importantly, they displayed a different localization pattern and very little overlap with endogenous human centromeres, as demonstrated by dCENP-A co-staining with human CENP-C (Figure S7D).

Both cell lines were transiently transfected with CAL1-GFP-LacIw to initiate dCENP-A loading at the LacO arrays. In addition, dCENP-A-SNAP was transfected to monitor dCENP-A *de novo* loading using the SNAP-tag technology (Dunleavy et al., 2012, Jansen et al., 2007, Lidsky et al., 2013, Schuh et al., 2007) and TetR-Myc to visualize the LacO site (Figure 7A). After 2 days of growth a subset of cells from both cell lines were subjected to IF and targeting of the GFP and SNAP-fusion construct to the LacO was confirmed in > 90% of the transfected cells (Figures 7A, 7B, and 7D). Remaining cells were treated with IPTG to remove CAL1-GFP-LacIw and SNAP-Cell Block to quench the existing dCENP-A-SNAP signal. Cells were washed and grown for additional 24 h in the presence of IPTG to allow synthesis of new dCENP-A-SNAP, which was tested for potential *de novo* loading at the LacO using tetramethylrhodamine (TMR)^∗^ labeling. As expected following the IPTG treatment, the GFP signal was no longer detectable at the LacO (Figure 7C). Importantly, whereas we could not observe new dCENP-A loading in the cell line stably expressing only dCENP-A, about 25% of cells expressing the three centromere proteins exhibited recruitment of new dCENP-A-SNAP-TMR^∗^ at LacO arrays (Figures 7C and 7D). dCENP-A inheritance was also tested over longer time periods (9 days) using an experimental setup in which both cell lines were first transiently transfected with GFP-LacI-dCENP-C (day 0; Figure S7A). After two days the triple-transgene-expressing cell line accumulated dCENP-A, CAL1-V5, and HA-dCENP-C at the LacO, as expected (Figures S7C and S7E). dCENP-A-mCherry was transfected after 7 days at which point cells had lost the transiently transfected GFP-LacI-CENP-C (Figure S7F). Similar to the IPTG/SNAP experiment, we found that new dCENP-A-mCherry was deposited when analyzed at day 9 in cells expressing all three *Drosophila* centromere factors but never for dCENP-A alone (Figure S7F). Note that due to the complex setup in this experiment the number of positive cells was not large enough to provide meaningful statistics and only allowed qualitative interpretation. We conclude that after temporary targeting CAL1 or dCENP-C to an ectopic chromosomal site in human cells, the *Drosophila* centromere proteins dCENP-C, CAL1, and dCENP-A are sufficient to create a self-propagating epigenetic loop to ensure centromeric dCENP-A inheritance from one cell generation to the next.

## Discussion

Understanding how epigenetic marks are propagated through the cell cycle to maintain chromatin identity is a key question in chromosome biology. The development of a heterologous system to investigate centromere inheritance in *Drosophila* was used to dissect step by step the roles of the three *Drosophila* centromere components dCENP-A, dCENP-C, and CAL1 in this process. It should be noted that this is an artificial system and does not reflect the endogenous protein levels or the complex organization of an endogenous *Drosophila* centromere. However, despite these drawbacks and unlike *in vitro* approaches, heterologous host cells can provide a physiological environment for reconstituting epigenetic inheritance through their ability to cycle, undergo replication, transcription, and supply important general players in these processes like the facilitates chromatin transcription (FACT)-complex or kinases (Chen et al., 2015, McKinley and Cheeseman, 2016). Providing a high level of control and reproducibility, it allowed us to successfully confirm all previously reported protein interactions, thereby lending strong support for the biological relevance of the new findings reported in this study.

We found that the CATD is partially conserved between *Drosophila* and humans as the dCATD is larger and required the majority of the histone-fold domain. As observed for humans, we have also shown that the dCATD of dCENP-A is similar to the domain recognized by the dCENP-A chaperone CAL1.

Importantly, our findings shed light on the mechanism of dCENP-C recruitment to chromatin, allowing us to close the epigenetic loop and propose a model for centromere propagation (Figure 7E). For dCENP-A loading, dCENP-C plays the role of the recruiting factor: a dCENP-C dimer recruits CAL1, likely in the form of a dimer in complex with 2× (dCENP-A/H4). Although the monomeric N-terminal fragment of CAL1 can form a trimeric complex with 1× dCENP-A/H4, we observe that CAL1’s ability to oligomerize is necessary for incorporation of dCENP-A into chromatin, thus suggesting that a dCENP-A/H4 tetramer is required for nucleosome formation. Although a plasmid supercoiling assay previously demonstrated that the subregion (1-160) of CAL1 lacking the self-association domain is sufficient for dCENP-A nucleosome assembly activity *in vitro* (Chen et al., 2014), excess of the dCENP-A/H4/CAL1 complex under these conditions might be sufficient to overcome the self-association requirement observed *in vivo*. A role for dCENP-C as a stable platform for CAL1/dCENP-A recruitment is further supported by experiments, indicating that dCENP-C is stably bound to centromeres (Lidsky et al., 2013, Mellone et al., 2011).

dCENP-C recruitment and maintenance at centromeres remain poorly understood. Here, we demonstrate that LacO-tethered CAL1 efficiently recruits dCENP-C in the presence of dCENP-A. Interestingly, although dCENP-C dimerization is required to interact with CAL1, a non-self-associating, monomeric fragment of CAL1 is sufficient to bind a dCENP-C dimer. Importantly, we find the stoichiometry of a CAL1:dCENP-C trimeric complex to be 1:2, which could explain the paradox of how CAL1 is able to bind a centromere-associated dCENP-C, yet also recruit new dCENP-C. These findings support an interesting hypothesis, in which one subunit of a CAL1 dimer interacts with a chromatin-bound dCENP-C dimer, while the other is available for recruiting a new soluble dCENP-C dimer (Figure 7E). This turns CAL1-mediated dCENP-C loading into the shortest possible epigenetic loop, were “reader” and “recruiter” are composed within the same protein.

CAL1 is unlikely to be required for dCENP-C maintenance; as in contrast to dCENP-C, it was shown to be very dynamic at centromeres (Lidsky et al., 2013, Mellone et al., 2011). In addition, we demonstrate that CAL1 does not stably associate with nucleosomal dCENP-A, suggesting that CAL1 dissociates from the chromatin after dCENP-A loading. Significant enhancement of dCENP-C localization at the LacO by the presence of dCENP-A nucleosomes instead suggests that dCENP-C maintenance might involve interaction with chromatin bound dCENP-A, similar to the human situation (Guse et al., 2011, Kato et al., 2013). This model also explains why dCENP-C was found to be dependent on both CAL1 and dCENP-A for its centromere targeting (Erhardt et al., 2008, Goshima et al., 2007, Schittenhelm et al., 2010). In contrast to previous reports using a two-hybrid approach (Schittenhelm et al., 2010), we do detect direct, albeit weak interactions between dCENP-C and dCENP-A (Figures 3I and 5B). Our finding that nucleosomal dCENP-A is required for dCENP-C recruitment suggests that these direct interactions occur most robustly on assembled dCENP-A chromatin, which could have been missed due to the experimental setup of the two-hybrid assay. Complementing the direct interaction with dCENP-A, dCENP-C’s stable chromatin binding could also involve its affinity to DNA through its adenine-thymine (AT) hook. Indeed, it was reported in humans, rats, and yeast that CENP-C has a DNA-binding property (Cohen et al., 2008, Politi et al., 2002, Sugimoto et al., 1994). CAL1 contains activities of both HJURP and the Mis18 complex through its ability to act as the chaperone for dCENP-A and bind centromeric dCENP-C, respectively. In addition, CAL1 also functions in the recruitment of dCENP-C to the centromere and acts as a potential loading factor. Interestingly, its human functional homolog HJURP was also shown to recruit a C-terminal fragment of hCENP-C (Tachiwana et al., 2015); so despite the poor protein conservation, our study suggests a general conserved mechanism for centromere inheritance in these organisms. Finally, we demonstrated that the epigenetic inheritance of dCENP-A can be “kick-started” by transiently targeting the *Drosophila* centromere factors CAL1 or dCENP-C to ectopic LacO regions in human cells stably expressing all three *Drosophila* transgenes dCENP-A, dCENP-C, and CAL1.

In summary, we identified CAL1 in conjunction with dCENP-A nucleosomes as a loader for dCENP-C, thereby closing the epigenetic loop for epigenetic dCENP-A propagation and demonstrated that combining these three centromere factors is sufficient to inherit *Drosophila* centromere identity.

## STAR★Methods

### Key Resources Table

**Table:** 

REAGENT or RESOURCE	SOURCE	IDENTIFIER
**Antibodies**		

rat anti-HA (3F10)	Roche	Cat# 3F10; RRID:AB_2314622
rabbit anti-hCENP-C	Black lab	N/A
mouse anti-hCENP-A	Abcam	Cat# ab13939; RRID:AB_300766
rabbit anti-dCENP-C	Lehner lab	N/A
rabbit anti-V5	Sigma-Aldrich	Cat# V8137; RRID:AB_261889
mouse anti-V5	Thermo Fisher	Cat# R960-25; RRID:AB_2556564
rabbit anti-Myc	Abcam	Cat# ab9106; RRID:AB_307014
chicken anti-dCENP-A	This paper	N/A
mouse anti-mCherry	Sacchani lab	N/A
mouse anti-GFP	Sacchani lab	N/A
mouse anti-HA 12CA5	Sigma-Aldrich	Cat#11583816001; RRID:AB_514505
rabbit anti-HA	Abcam	Cat# ab9110, RRID:AB_307019

**Bacterial and Virus Strains**		

ElectroMAX DH5α-E Competent Cells	ThermoFisher Scientific	11319019

**Chemicals, Peptides, and Recombinant Proteins**		

dCENP-A(101-225)	This paper	pET3a
dH4	Karolin Luger	pET3a
Human H4	This paper	pEC-K-3C-His-GST
CAL1(1-160)	This paper	pEC-K-3C-His-GST
CAL1 (841-979)	This paper	pEC-K-3C-His-GST
dCENP-C (1264-1411)	This paper	pEC-K-3C-His-GST

**Experimental Models: Cell Lines**		

U2OS-LacO (female)	S. Janicki lab	
U2OS-LacO- CAL1V5_T2A_ dCENP-A_T2A_ HAdCENP-C (clone 17)	This paper	N/A
U2OS-LacO-dCENP-A (clone4)	This paper	N/A
Schneider S2 cells (L2-4; male)	Botchan lab	N/A

**Oligonucleotides**		

αSatCh17-f2: CGTTGGAAACGGGATAATTTCAGCTGACTA	This paper	N/A
αSatCh17-r2: CACAGAGTGGTCCAAATATCCACTTGTAGA	This paper	N/A
αSatCh21-f2: GGCCTTTCATAGAGCAGGTTTGAAACACTC	This paper	N/A
αSatCh21-r2: CAAATATCCACTTGCAGATTCCACAAAAAG	This paper	N/A
LacO_F TAGAGGCGCCGAATTCCA	Mendiburo et al., 2011	N/A
LacO_R2 ATCCGCTCACAATTCCAC	Mendiburo et al., 2011	N/A

**Recombinant DNA**		

pDS47_dCENP-A_HA	This paper	N/A
pDS47_H3_HA	This paper	N/A
pDS47_H3^L1α2^_HA	This paper	N/A
pDS47_dCENP-A^C^_HA	This paper	N/A
pDS47_dCENP-A ^α2+C^_HA	This paper	N/A
pDS47_H3 ^L1α2+C^ _HA	This paper	N/A
pDS47_H3 ^α2+C^ _HA	This paper	N/A
pDS47_H3 ^C^ _HA	This paper	N/A
pDS47_dCENP-A ^L1α2^ _HA	This paper	N/A
pDS47_dCENP-A ^L1α2+C^ _HA	This paper	N/A
pDS47_H3 ^L1+C^ _HA	This paper	N/A
pDS47_H3 ^L1α2+C(223)^ _HA	This paper	N/A
pDS47_H3 ^L1α2+C(222)^ _HA	This paper	N/A
pDS47_H3 ^L1α2+C(219)^ _HA	This paper	N/A
pDS47_H3 ^L1α2+C(218)^ _HA	This paper	N/A
pDS47_H3 ^L1α2+C(206)^ _HA	This paper	N/A
pMT_CAL1_HA	This paper	N/A
pMT_CAL1(NT)_HA	This paper	N/A
pMT_CAL1(CT)_HA	This paper	N/A
pN2_CMV_dCENP-A_GFPLacI	This paper	N/A
pN2_CMV _H3_ GFPLacI	This paper	N/A
pN2_CMV _H3^L1α2^_ GFPLacI	This paper	N/A
pN2_CMV _dCENP-A^C^_ GFPLacI	This paper	N/A
pN2_CMV _dCENP-A ^α2+C^_ GFPLacI	This paper	N/A
pN2_CMV _H3 ^L1α2+C^ _ GFPLacI	This paper	N/A
pN2_CMV _H3 ^α2+C^ _ GFPLacI	This paper	N/A
pN2_CMV _H3 ^C^ _ GFPLacI	This paper	N/A
pN2_CMV _dCENP-A ^L1α2^ _ GFPLacI	This paper	N/A
pN2_CMV _dCENP-A ^L1α2+C^ _ GFPLacI	This paper	N/A
pN2_CMV _H3 ^L1+C^ _ GFPLacI	This paper	N/A
pN2_CMV _ GFPLacI_dCENP-C(CT)	This paper	N/A
pN2_CMV _ GFPLacI	This paper	N/A
pN2_CMV _ GFPLacI_HJURP	This paper	N/A
pN2_CMV_CAL1_GFPLacI	This paper	N/A
pN2_CMV _ GFPLacI_dCENP-C	This paper	N/A
pN2_CMV_CAL1_V5	This paper	N/A
pN2_CMV_dCENP-A_V5	This paper	N/A
pN2_CMV_tetR_V5	This paper	N/A
pN2_CMV _dCENP-A^C^_V5	This paper	N/A
pN2_CMV _dCENP-A^C^_V5	This paper	N/A
pN2_CMV _ HA_dCENP-C	This paper	N/A
pN2_CMV_CAL1_HA	This paper	N/A
pN2_CMV_dCENP-A_HA	This paper	N/A
pN2_CMV _H3_ HA	This paper	N/A
pN2_CMV_CAL1(NT)_HA	This paper	N/A
pN2_CMV_CAL1(CT)_HA	This paper	N/A
pN2_CMV_CAL1(MID)_HA	This paper	N/A
pN2_CMV_CAL1(1-160)_HA	This paper	N/A
pN2_CMV_CAL1(101-407)_HA	This paper	N/A
pN2_CMV _ HA_dCENP-C(NT)	This paper	N/A
pN2_CMV _ HA_dCENP-C(CT)	This paper	N/A
pN2_CMV _ HA_dCENP-C(MID)	This paper	N/A
pN2_CMV _ HA_dCENP-C(1009-1263)	This paper	N/A
pN2_CMV _ HA_dCENP-C(1264-1411)	This paper	N/A
pN2_CMV _ HA_dCENP-C(1264-1411)L1357E-L1406E	This paper	N/A
pN2_CMV _ HA_dCENP-C_L1357E-L1406E	This paper	N/A
pN2_CMV_HA_hCENP-C	This paper	N/A
pN2_CMV_hCENP-A_HA	This paper	N/A
pcDNA3_CMV_HA_HJURP	This paper	N/A
pN2_CMV_CAL1(NT)_ GFPLacIw	This paper	N/A
pN2_CMV_CAL1(1-160)_ GFPLacIw	This paper	N/A
pN2_CMV _ GFPLacIw	This paper	N/A
pN2_CMV_dCENP-A(Y187A-L188Q)_GFPLacIw	This paper	N/A
pN2_CMV _dCENP-A^C^_ GFPLacIw	This paper	N/A
pN2_CMV_dCENP-A_GFPLacIw	This paper	N/A
pN2_CMV_CAL1_ GFPLacIw	This paper	N/A
pN2_CMV_ GFPLacIw_dCENP-C	This paper	N/A
pN2_CMV_tetR_Myc	This paper	N/A
pN2_CMV_dCENP-A_SNAP	This paper	N/A
pN2_CMV_dCENP-A_mCherry	This paper	N/A
pN2_CMV_CAL1V5_T2A_ dCENP-A_T2A_ HAdCENP-C	This paper	N/A
pET3a_dCENP-A(101-225)	This paper	N/A
pET3a_dH4	Karolin Luger	N/A
pEC-K-3C-His-GST_hH4	This paper	N/A
pEC-K-3C-His-GST_CAL1(1-160)	This paper	N/A
pEC-K-3C-His-GST_CAL1 (841-979)	This paper	N/A
pEC-K-3C-His-GST_dCENP-C (1264-1411)	This paper	N/A

**Software and Algorithms**		

ImageJ		
Prism	GraphPad	Prism 8.0

**Other**		

Adobe Illustrator	Adobe	CC 2019
Adobe Photoshop	Adobe	CC 2019
softWoRx Explorer Suite 2.0	Applied Precision	2.0

### Lead Contact and Materials Availability

The reagents created are freely available and no unique reagents were generated. Further information and requests for resources and reagents should be directed to and will be fulfilled by the Lead Contact, Patrick Heun (Patrick.Heun@ed.ac.uk).

### Experimental Model and Subject Details

#### Cell Culture

U2OS cells (female) containing 200 copies of an array of 256 tandem repeats of the 17 bp LacO sequence on chromosome 1 (gift from S. Janicki; Janicki et al., 2004) were cultured in DMEM supplemented with 10% FBS, 100 U/ml penicillin, 100 μg/ml streptomycin and selected with 100 μg/ml Hygromycin B at 37°C in a humidified incubator with 5% CO2. Schneider S2 cells (male) were grown at 25°C in Schneider’s *Drosophila* medium (Serva) supplemented with 10% FBS (Sigma-Aldrich) and antibiotics (300 μg/ml penicillin, 300 μg/ml streptomycin, and 750 μg/ml amphotericin B).

### Method Details

#### Transfection

U2OS cells were transfected with FuGENE HD Transfection Reagent (Promega) and Schneider S2 cells with XtremeGENE DNA transfection reagent (Roche).

#### Plasmids

All *Drosophila* expression vectors used in this study were constructed in a pMT/V5-6his vector or a pDS47 vector. All mammalian expression vectors used in this study were constructed in a pN2-CMV vector.

As originally described (Straight et al., 1996), the LacI protein used in this study contained an 11-amino-acid-C-terminal truncation to prevent tetramer formation and to avoid artifactual linkage of daughter chromatids during mitosis when targeted to the chromosomally-integrated LacO array. This LacI protein is insensitive to IPTG treatment. Therefore, for all experiments in which the LacI fusion needed to be displaced from the LacO array, a double mutant version of LacI, termed “LacI weak” was used. This mutant was derived from a construct obtained from Loiodice et al. (2014) and comprises three mutations toward the N terminus of the protein (G58D, Q60GGG and T68S) in addition to the C-terminal truncation.

For the cloning of the vector with multiple genes under the same promoter, we used the Golden Gate assembly method as described in Engler and Marillonnet (2014) and Szymczak et al. (2004) and the genes were separated with T2A or P2A sequences to create a self-cleaving peptide.

For expression of the recombinant proteins in SEC-MALS and gel filtration assays, dCENP-A and dH4 were cloned into a pET3a vector, CAL1(1-160) into a pEC-K-3C-His-GST vector, dCENP-C and hH4 into a pEC-K-3C-His vector, all containing a T7 promoter.

#### Immunofluorescence

Cells were harvested 48 h (for U2OS-LacO cells) or 72 h (for S2 cells) after transfection and fixed for 10 min in 3.7% formaldehyde in PBS and 0.1% Triton X-100. Primary antibodies were incubated overnight at 4°C at the following dilutions or concentrations: rat anti-HA (1:20; clone 3F10), rabbit anti-hCENP-C (1:100; B. Black; Bassett et al., 2010), mouse anti-hCENP-A, rabbit anti-dCENP-C (1:100; a gift from C. Lehner, University of Zurich, Zurich, Switzerland; Heeger et al., 2005), rabbit anti-V5 (1:500), mouse anti-V5 (1:100; ), rabbit anti-Myc (1:100), chicken anti-dCENP-A (1:100), mouse anti-mCherry (1:15; kindly provided by S. Sacchani). Goat secondary antibodies conjugated to Alexa Fluor 488, Alexa Fluor 555 or Alexa Fluor 647 (Invitrogen) were incubated for 1 h at room temperature at a dilution of 1:100. DNA was counterstained with DAPI at 5 μg/ml.

For experiments involving IPTG treatment on U20S-LacO cells, cells were treated 48 h after transfection with 15 mM IPTG (Sigma-Aldrich) for 1h and processed for immuno-fluorescence, as described earlier in this section.

Cells transfected with CENP-A-SNAP were labeled with TMR-Star by incubating a subset of cells after 48 hr (day 2) directly with 2 μM SNAP-Cell TMR-Star (New England BioLabs) containing media for 15 min, washed with PBS and harvested for IF. Another subset was treated with 20 μM SNAP-Cell® Block (New England Biolabs) and IPTG-containing media for 30 min, washed with phosphate-buffered saline (PBS) and left for 24 hours in fresh IPTG-medium. Newly synthesized CENP-A-SNAP was labeled by incubating cells on day 3 with 2 μM SNAP-Cell TMR-Star (New England BioLabs) containing media for 15 min, washed with PBS and harvested for IF.

#### Western Blotting

U2OS and S2 whole cell extracts were loaded on 4%–20% Tris-Glycine gels and blotted on Amersham Protran 0.2 NC nitrocellulose membranes. Blots were blocked in 5% milk/PBS–Tween and incubated with mouse anti-V5 1:1000 or mouse anti-GFP 1:5000 (clone 71, kind gift from D. van Essen) or anti-mouse HA.

#### SEC MALS

Size-exclusion chromatography (ÄKTAMicro, GE Healthcare) coupled to UV, static light scattering, and refractive index detection (Viscotek SEC-MALS 20 and Viscotek RI Detector VE3580; Malvern Instruments) was used to determine the absolute molecular mass of proteins and protein complexes in solution. Injections of 100 μl of 1-5 mg/ml material were run on a Superdex 200 10/300 Increase GL (GE Healthcare) size-exclusion column pre-equilibrated in 50 mM HEPES (pH 8.0), 150 or 300 mM NaCl, and 1 mM TCEP at 22°C with a flow rate of 0.5 ml/min. Light scattering, refractive index (RI), and A_280 nm_ were analyzed by a homo-polymer model (OmniSEC software, v5.02; Malvern Instruments) using the following para-meters for: *∂*A_280 nm_/*∂*c = 1.04 AU.ml.mg ^-1^ (Cal1 1-160), 0.70 AU.ml.mg ^-1^ (Cal1 1-160/H4/CID 101-end), 0.79 AU.ml.mg ^-1^ (Cal1 1-160/H4/CID 144-end), 0.75 AU.ml.mg ^-1^ (His CENP-C 1264-end), 0.89 AU.ml.mg^-1^ (CAL1_841-979_/CENP-C_1264-1411_), *∂*n/*∂*c = 0.185 mL g ^-1^ and buffer RI value of 1.335.)

The mean standard error in the mass accuracy determined for a range of protein–protein complexes spanning the mass range of 6–600 kDa is ± 1.9%.

#### ChIP Assay

U2OS cells were collected, fixed in 1% PFA for 15 minutes in rotation and subsequently quenched with 0.125 M glycine for 15 minutes in rotation. After quenching, the cells were lysed in 500μl of lysis buffer (1% SDS, 10mM EDTA 50mM Tris-HCl pH 8, protease inhibitor cocktail) on ice for 10 minutes. Lysates were sonicated four times for 15 minutes each in Bioruptor (H, 30’’ on / 30’’ off). Sonicated lysates were then diluted to 5 mL with dilution solution (0.01% SDS, 1% Tx100, 2mM EDTA, 20mM Tris-HCl pH 8, 150 mM NaCl, protease inhibitor cocktail), and used for antibody incubation. Rabbit IgG was used as a control and HA antibody. Samples were incubated overnight at 4°C in a rotating rotor. Protein A coupled magnetic beads (Biorad) were blocked overnight with 0.05% BSA, 2μg/ml of salmon sperm DNA in dilution solution. Antibody and control fractions were then incubated with the magnetic beads for 4 hours at 4°C in a rotating rotor, and subsequently washed with buffer 1 (0.1% SDS, 1% Tx100, 2mM Tris-HCl pH 8, 150mM NaCl), buffer 2 (0.1% SDS, 1% Tx100, 2mM Tris-HCl pH 8, 500mM NaCl), buffer 3 (1% NP40, 1% sodium deoxycholate, 10mM Tris-HCl pH 8, 1mM EDTA, 0.25mM LiCl), and finally with TE buffer. 10% CHELEX was added to the samples and then de-crosslinked at 95°C for 10 minutes, Proteinase K treated (2μg/ml) at 55°C for 30 minutes and inactivated at 95°C for 10 minutes. Supernatant was used for direct qPCR reaction (2μl).

#### Microscopy

Two microscopes were used to image the samples. With a microscope DeltaVision RT Elite, images were acquired as 45-50 z stacks of 0.2 μm increments using a 100 × oil immersion objective and a mono-chrome camera (CoolSNAP HQ; Photometrics) and deconvolved using softWoRx Explorer Suite (Applied Precision). With an Olympus confocal microscope FV1200, images were acquired as 10-20 z stacks of 0.42 μm increments using a 60 × oil immersion objective.

### Quantification and Statistical Analysis

#### Image Analysis

Quantification of fluorescence intensities was performed using ImageJ. For experiments with S2 cells, the mean fluorescence intensity of the protein of interest was measured at centromeres and subtracted from the average of the mean fluorescence intensities of three points randomly chosen in the nucleus (background), then normalized to the mean fluorescent intensity of centromeres (marked with anti dCENP-C or dCENP-A antibodies). For the experiments with U2OS-LacO cells, the mean fluorescence intensity of the protein of interest was measured at the LacO spot, subtracted from the mean fluorescence intensity in the nucleus (background) and normalized to the GFP (in case of GFP-LacI tagged proteins), V5 or Myc (tetR-V5 or tetR-Myc in experiments with IPTG treatment) mean fluorescence intensity of the corresponding LacO spot. A minimum of 20 cells were analyzed per biological replicate, and a minimum of two independent biological replicates were quantified per experiment.

#### Statistical Analysis

GraphPad Prism software was used for statistical analysis and graphical representations. Unpaired Student’s t tests were performed in Figures 1E, 3B, 3C, 3H, 3I, 5B, 5D, 6D, 6F, 7D, S5C, and S5D; one-way ANOVA with Dunnett’s post-test in Figures 1D, 2F, 4B, 4C, 4H, 5E, 6B, 6E, and S4D; and one-way ANOVA with Turkey’s post-test in Figures 2C, 2D, and S4B. Error bars represent mean ± SEM.

### Data and Code Availability

This study did not generate/analyze datasets/code.

## Supplemental Information

Document S1. Figures S1–S7

Document S2. Article plus Supplemental Information
