# Supplementary material for: Reconstituting Drosophila Centromere Identity in Human Cells
Source: Cell Rep. 2019 Oct 8;29(2):464–479.e5. doi: 10.1016/j.celrep.2019.08.067 (PMC6900781; doi:10.1016/j.celrep.2019.08.067)
Supplement: Document S1. Figures S1–S7 [file mmc1.pdf]

Cell Reports, Volume *29*

Supplemental Information

Reconstituting *Drosophila* Centromere Identity in Human Cells

Virginie Roure, Bethan Medina-Pritchard, Vasiliki Lazou, Luciano Rago, Eduard Anselm, Daniela Venegas, A. Arockia Jeyaprakash, and Patrick Heun

Roure_FigS1

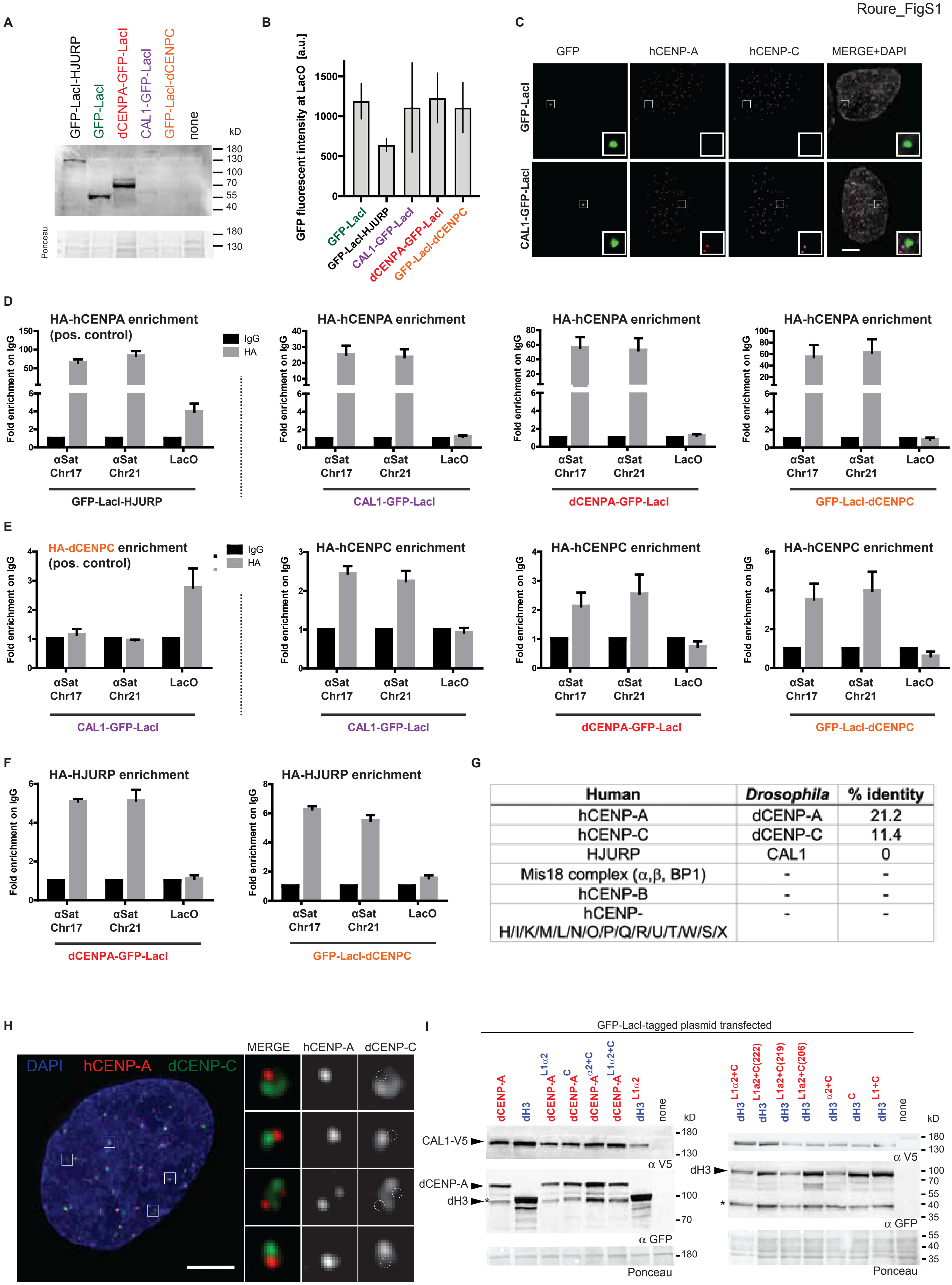

Human	*Drosophila*	% identity
hCENP-A	dCENP-A	21.2
hCENP-C	dCENP-C	11.4
HJURP	CAL1	0
Mis18 complex (α,β, BP1)	-	-
hCENP-B	-	-
hCENP-H/I/K/M/L/N/O/P/Q/R/U/T/W/S/X	-	-

Figure S1. The host system required for establishing human centromere identity does not interfere with the exogenous *Drosophila* centromere identity system, Related to Figure 2.

Expression levels of GFP-LacI-tagged transgenic constructs used in Figures 2B -2D were evaluated by (A) western blotting with α-GFP antibody and (B) measuring GFP-fluorescence intensity at the LacO site using microscopy. Note that although the CAL1 and dCENP-C GFP-LacI display GFP-fluorescence levels similar to dCENP-A or GFP-LacI, they cannot be detected by western blot, likely due to lysate instability. (C) Representative IF images of hCENP-A and hCENP-C recruitment to the LacO arrays byCAL1-GFP-LacI and the control GFP-LacI in U2OS cells. (D-F) Chromatin immunoprecipitation (ChIP) assays were performed to analyse enrichment of human centromere factors hCENP-A, hCENP-C and HJURP at human centromeres of chromosome 17 (alpha satellite 17) and chromosome 21 (alpha satellite 21) and at LacO arrays (LacO) in U2OS cells. (D) HA-tagged hCENPA was immunoprecipitated when co-expressed with GFP-lacI-HJURP (positive control), CAL1-GFP-lacI, dCENPA-GFP-lacI, or GFP-lacI-dCENPC. (E) ChIP for HA-tagged dCENPC (positive control) and HA-tagged hCENP-C upon tethering of the GFP-LacI-tagged *Drosophila* factor CAL1, dCENP-A or dCENP-C to LacO arrays. (F) ChIP for HA-tagged HJURP upon tethering of GFP-LacI-tagged dCENP-A and dCENP-C to LacO arrays. All enrichments are compared to IgG controls, two independent experiments were performed. (G) Homologies between Human and *Drosophila* centromere proteins. (H) IF image of a U2OS cell co-stained with transgenic dCENP-C (green) and human CENP-C (red) as centromere counterstain. Insets show magnification of the boxed regions. Scale bar, 5µm. (I) Expression levels of GFP-LacI-tagged chimeras were evaluated by Western blotting with α-V5 or α-GFP to detect Cal1 or dCENPA/dH3 respectively. Note that efficiency of recruitment does not correlate with protein levels.

Roure_FigS2

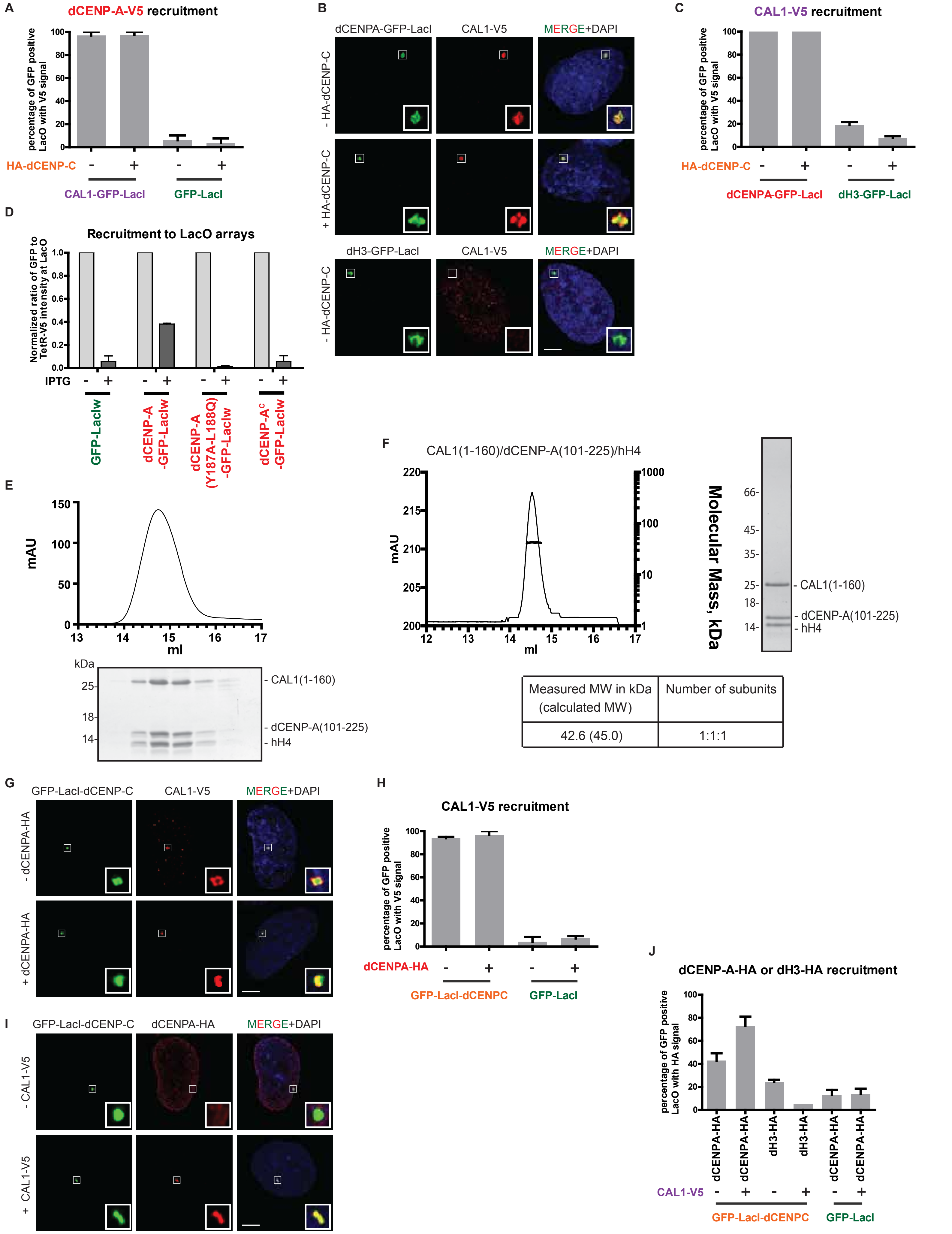

Measured MW in kDa (calculated MW)	Number of subunits
42.6 (45.0)	1:1:1

Figure S2. dCENP-C recruits the CAL1/dCENP-A complex to chromatin, Related to Figure 3.

(A) Quantification of the percentage of GFP-positive cells that recruit dCENP-A-V5 at LacO upon tethering of CAL1-GFP-LacI or the control GFP-LacI, +/- HA-dCENP-C (for signal intensities see Figure 3B). (B) Representative IF images of CAL1-V5 recruitment to the LacO arrays by GFP-LacI-tagged dCENP-A and dH3, +/- HA-dCENP-C, in U2OS cells. (C) Quantification of the percentage of GFP-positive cells that recruit CAL1-V5 at LacO upon tethering of GFP-LacI-tagged dCENP-A or dH3, +/- HA-dCENP-C (for signal intensities see Figure 3C). (D) Quantitation of normalized mean intensities at LacO of the indicated WT dCENP-A and chimeras fused to GFP-LacIw, +/- IPTG treatment. (E) SEC profiles and respective SDS-PAGE analysis of CAL1(1-160) in complex with dCENP-A(101-225) and hH4 complex separated on a S200 increase 10/300. (F) SEC-MALS experiments performed with recombinant CAL1 (1-160) in complex with dCENP-A (101-225) and human H4. Elution volume (ml, x-axis) is plotted against absorption at 280 nm (mAu, left y-axis) and molecular mass (kDa, right y-axis). Tables show measure molecular weight (MW) and calculated MW. Mw/Mn = 1.001. (G, I) Representative IF images of the recruitment to LacO arrays by GFP-LacI-dCENP-C of CAL1-V5, +/- dCENP-A-HA (G), and dCENP-A-HA, +/- CAL1-V5 (I), in U2OS cells. Insets show magnification of the boxed regions. Scale bar, 5µm. (H) Quantification of the percentage of GFP-positive cells that recruit CAL1-V5 at LacO upon tethering of GFP-LacI-tagged dCENP-C or GFP-LacI alone, +/- dCENP-A-HA (for signal intensities see Figure 3H). (J) Quantification of the percentage of GFP-positive cells that recruit dCENP-A-HA or dH3-HA at LacO upon tethering of GFP-LacI-tagged dCENP-C or GFP-LacI alone, +/- dCAL1-V5 (for signal intensities see Figure 3I).

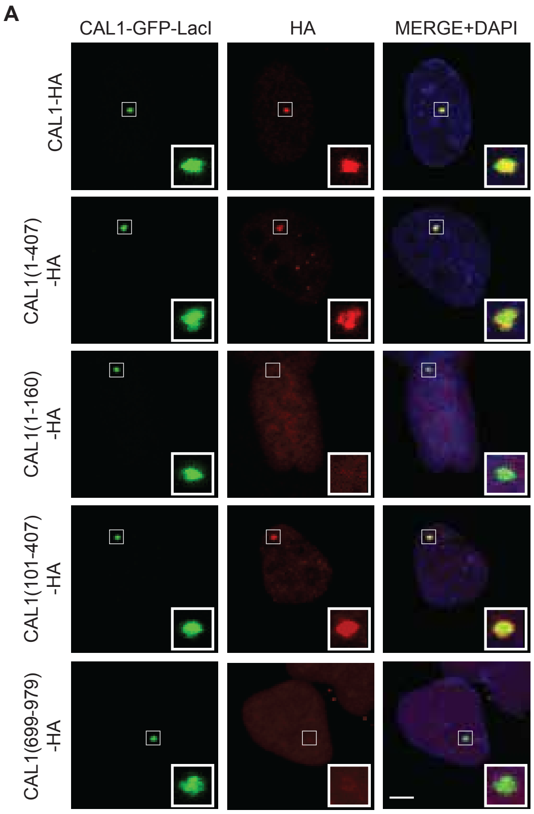

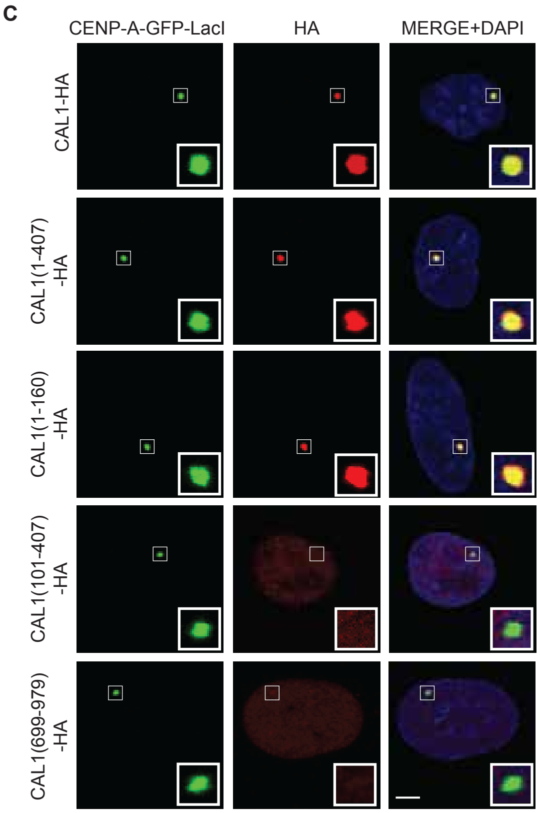

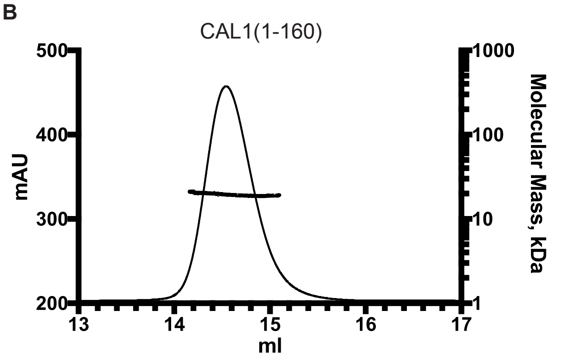

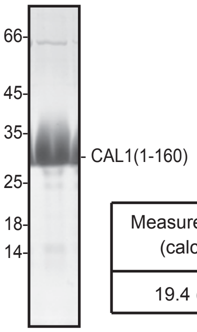

Measured MW in kDa (calculated MW)	Number of subunits
19.4 (18.6)	1

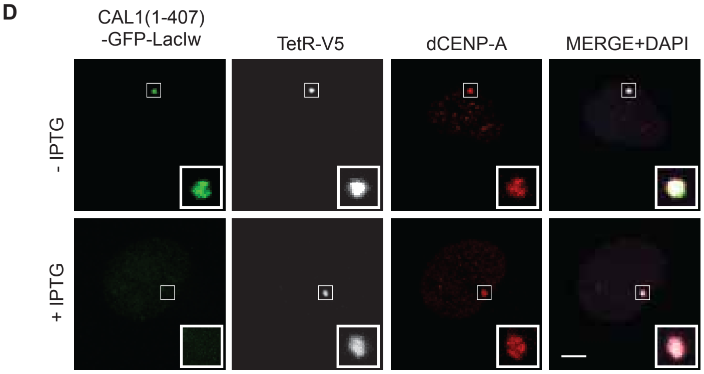

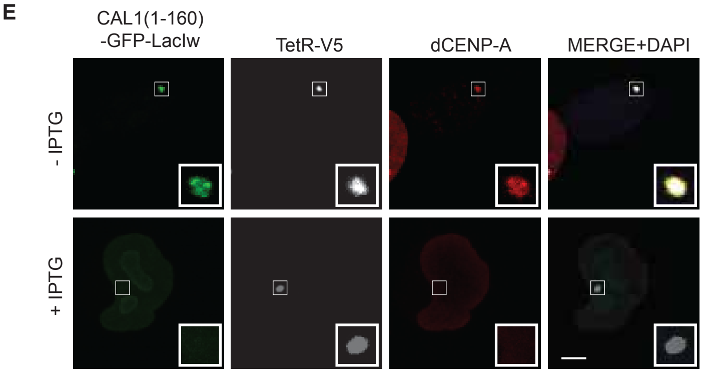

Figure S3. CAL1 self-association is not required for binding but for deposition of dCENP-A, Related to Figure 4.

(A, C) Representative IF images of HA-tagged CAL1 and CAL1 fragment recruitment to the LacO arrays by CAL1-GFP-LacI (A) and dCENP-A-GFP-LacI (C) in U2OS cells. (B) SEC MALS experiments performed with recombinant CAL1(1-160). Elution volume (ml, x-axis) is plotted against absorption at 280 nm (mAu, left y-axis) and molecular mass (kDa, right y-axis). Tables show measure molecular weight (MW) and calculated MW. Mw/Mn = 1.001. (D, E) Representative IF images of dCENP-A recruitment to the LacO arrays by CAL1(1-407) (D) and CAL1(1-160) (E) fused to GFP-LacIw, +/- IPTG treatment in U2OS cells. Insets show magnification of the boxed regions. Scale bar, 5µm.

Roure_FigS4

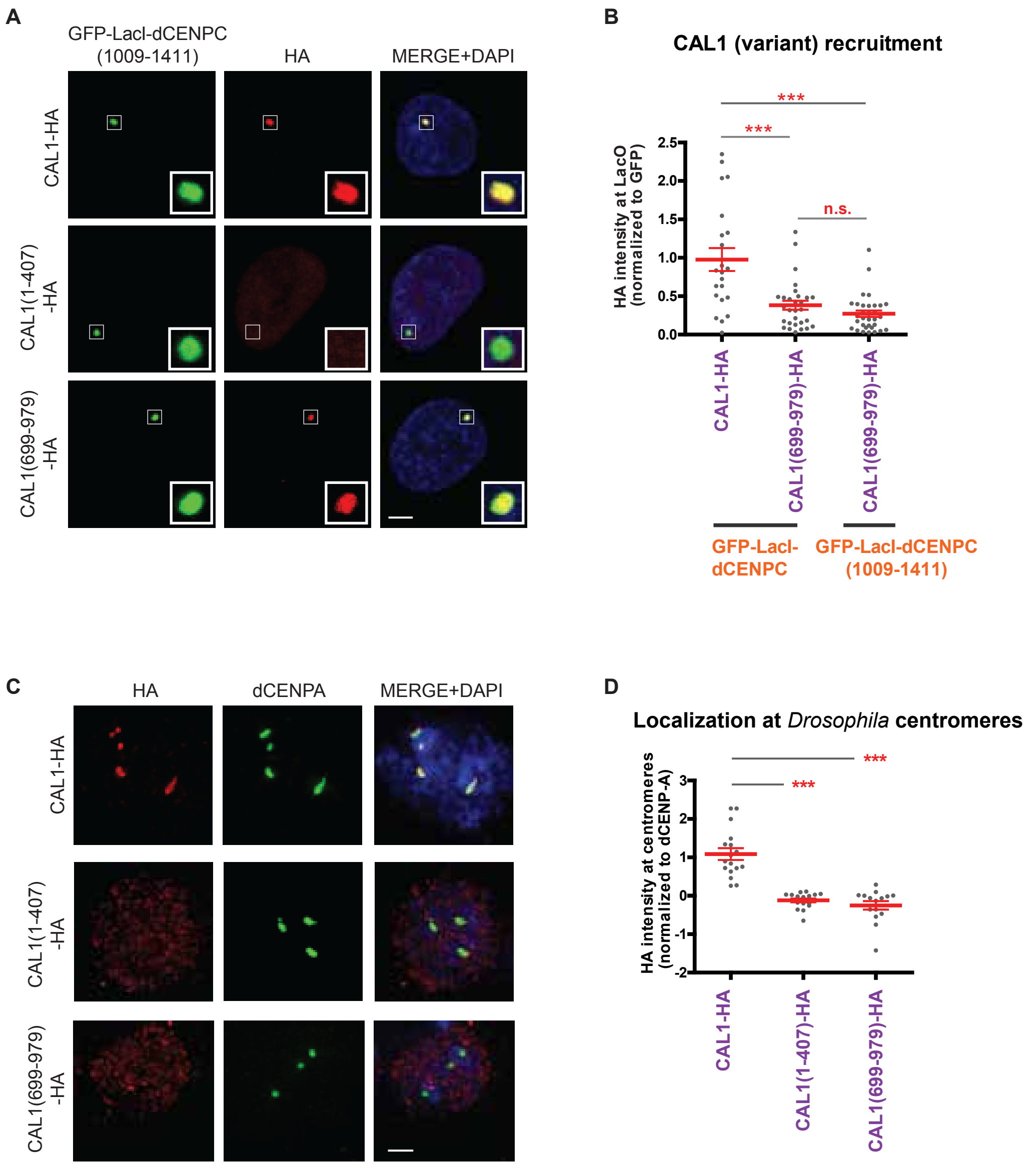
A
GFP-LacI-dCENPC (1009-1411)
HA
MERGE+DAPI
CAL1-HA
CAL1(1-407) -HA
CAL1(699-979) -HA
B
CAL1 (variant) recruitment
HA intensity at LacO (normalized to GFP)
***
***
n.s.
0.0
0.5
1.0
1.5
2.0
2.5
CAL1-HA
CAL1(699-979)-HA
CAL1(699-979)-HA
GFP-LacI-dCENPC
GFP-LacI-dCENPC (1009-1411)
C
HA
dCENPA
MERGE+DAPI
CAL1-HA
CAL1(1-407) -HA
CAL1(699-979) -HA
D
Localization at Drosophila centromeres
HA intensity at centromeres (normalized to dCENP-A)
***
***
-2
-1
0
1
2
3
CAL1-HA
CAL1(1-407)-HA
CAL1(699-979)-HA

Figure S4. Full length CAL1 is more efficiently recruited to dCENP-C then its C-terminal fragment, Related to Figure 4.

(A) Representative IF images of HA-tagged CAL1 and CAL1 fragment recruitment to the LacO arrays by GFP-LacI-dCENP-C(1009-1411) in U2OS cells. (B) Quantitation of normalized HA-tagged CAL1 and CAL1(699-979) mean intensities at LacO upon tethering of GFP-LacI-tagged dCENP-C and dCENP-C (1009-1411). (C) Representative IF images of HA-tagged CAL1 and CAL1 fragment expression patterns in S2 *Drosophila* cells. dCENP-A marks *Drosophila* centromeres. (D) Quantitation of normalized mean intensities of the indicated CAL1 and CAL1 fragments at centromeres. Insets show magnification of the boxed regions. Scale bar, 5μm. Error bars show SEM (*** $P < 0.001$; (n.s.) as not significant).

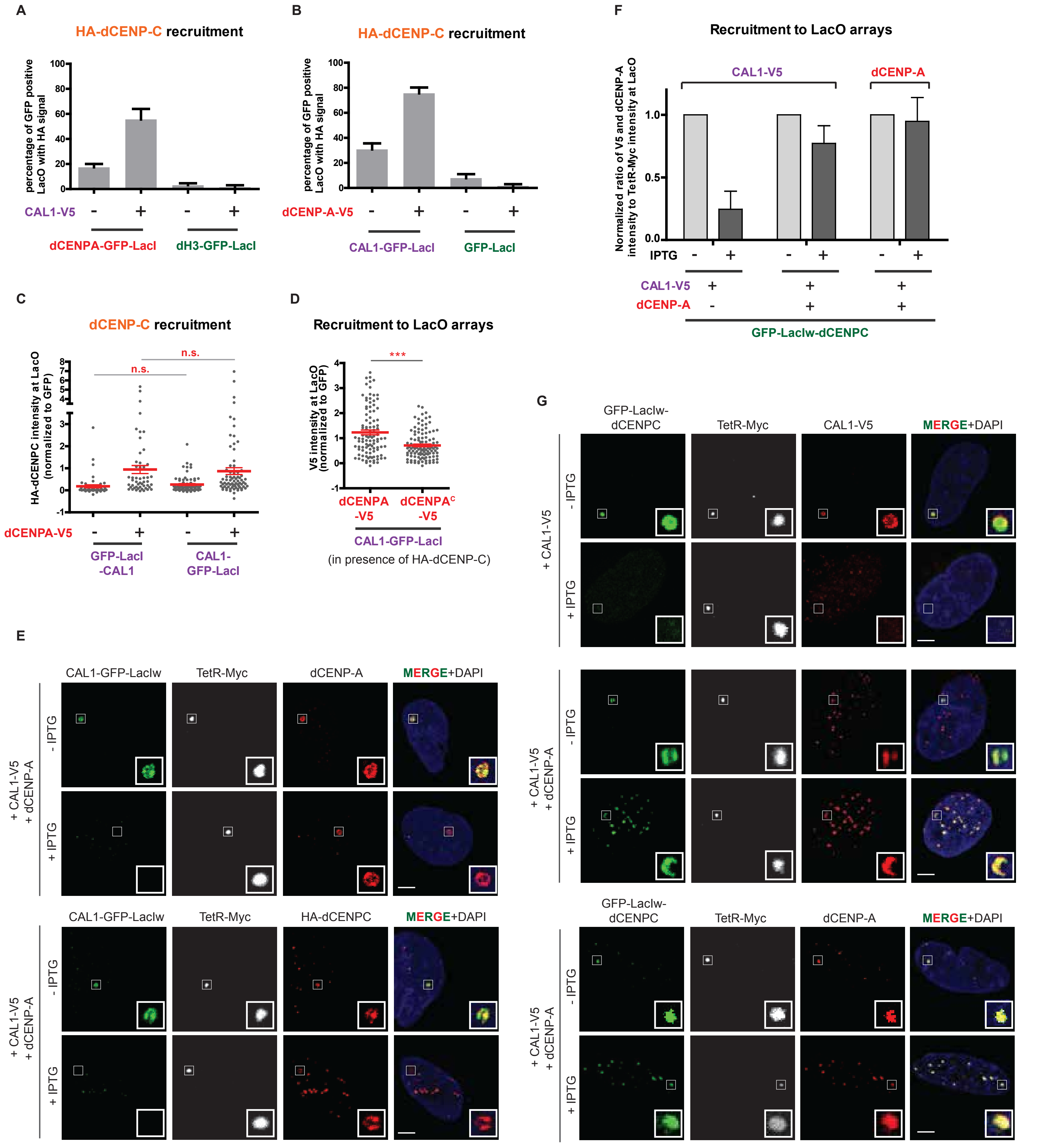
A
HA-dCENP-C recruitment
percentage of GFP positive LacO with HA signal
CAL1-V5
dCENPA-GFP-LacI
dH3-GFP-LacI
B
HA-dCENP-C recruitment
dCENP-A-V5
CAL1-GFP-LacI
GFP-LacI
C
dCENP-C recruitment
HA-dCENPC intensity at LacO (normalized to GFP)
n.s.
n.s.
dCENPA-V5
GFP-LacI -CAL1
CAL1-GFP-LacI
D
Recruitment to LacO arrays
V5 intensity at LacO (normalized to GFP)
***
dCENPA -V5
dCENPA^C -V5
CAL1-GFP-LacI
(in presence of HA-dCENP-C)
F
Recruitment to LacO arrays
Normalized ratio of V5 and dCENP-A intensity to TetR-Myc intensity at LacO
CAL1-V5
dCENP-A
IPTG
CAL1-V5
dCENP-A
GFP-LacIw-dCENPC
E
CAL1-GFP-LacIw
TetR-Myc
dCENP-A
MERGE+DAPI
+ CAL1-V5 + dCENP-A
- IPTG
+ IPTG
CAL1-GFP-LacIw
TetR-Myc
HA-dCENPC
MERGE+DAPI
G
GFP-LacIw-dCENPC
TetR-Myc
CAL1-V5
MERGE+DAPI
+ CAL1-V5
+ CAL1-V5 + dCENP-A
GFP-LacIw-dCENPC
TetR-Myc
dCENP-A
MERGE+DAPI

Figure S5. dCENP-C is loaded by CAL1 to the lacO arrays containing dCENP-A nucleosomes, Related to Figure 5.

(A) Quantification of the percentage of GFP-positive cells that recruit HA-dCENP-C at LacO upon tethering of GFP-LacI-tagged dCENP-A or dH3, +/- dCAL1-V5 (for signal intensities see Figure 5B). (B) Quantification of the percentage of GFP-positive cells that recruit HA-dCENP-C at LacO upon tethering of CAL1-GFP-LacI or GFP-LacI, +/- dCENP-A-V5 (for signal intensities see Figure 5D). (C) Quantitation of normalized HA-dCENP-C mean intensity at LacO upon tethering of CAL1 fused to GFP-LacI on its N-terminus (GFP-LacI-CAL1) or C-terminus (CAL1-GFP-LacI), +/- dCENP-A-V5. (D) Quantitation of normalized V5-tagged dCENP-A and dCENP-A^{C} mean intensities at LacO upon tethering of CAL1-GFP-LacI in presence of HA-dCENP-C. (E) Representative IF images of dCENP-A and HA-dCENP-C recruitment to the LacO arrays by CAL1-GFP-LacIw, +/- IPTG treatment in U2OS cells. (F) Quantitation of normalized CAL1-V5 and dCENP-A mean intensities at LacO upon tethering of GFP-LacIw-dCENP-C, +/-dCENP-A, +/- IPTG treatment. (G) Representative IF images of CAL1-V5 and dCENP-A recruitment to the LacO arrays by GFP-LacIw-dCENP-C, +/-dCENP-A, +/- IPTG treatment in U2OS cells. Insets show magnification of the boxed regions. Scale bar, 5µm. Error bars show SEM (*** $P < 0.001$; (n.s.) as not significant).

Roure_FigS6

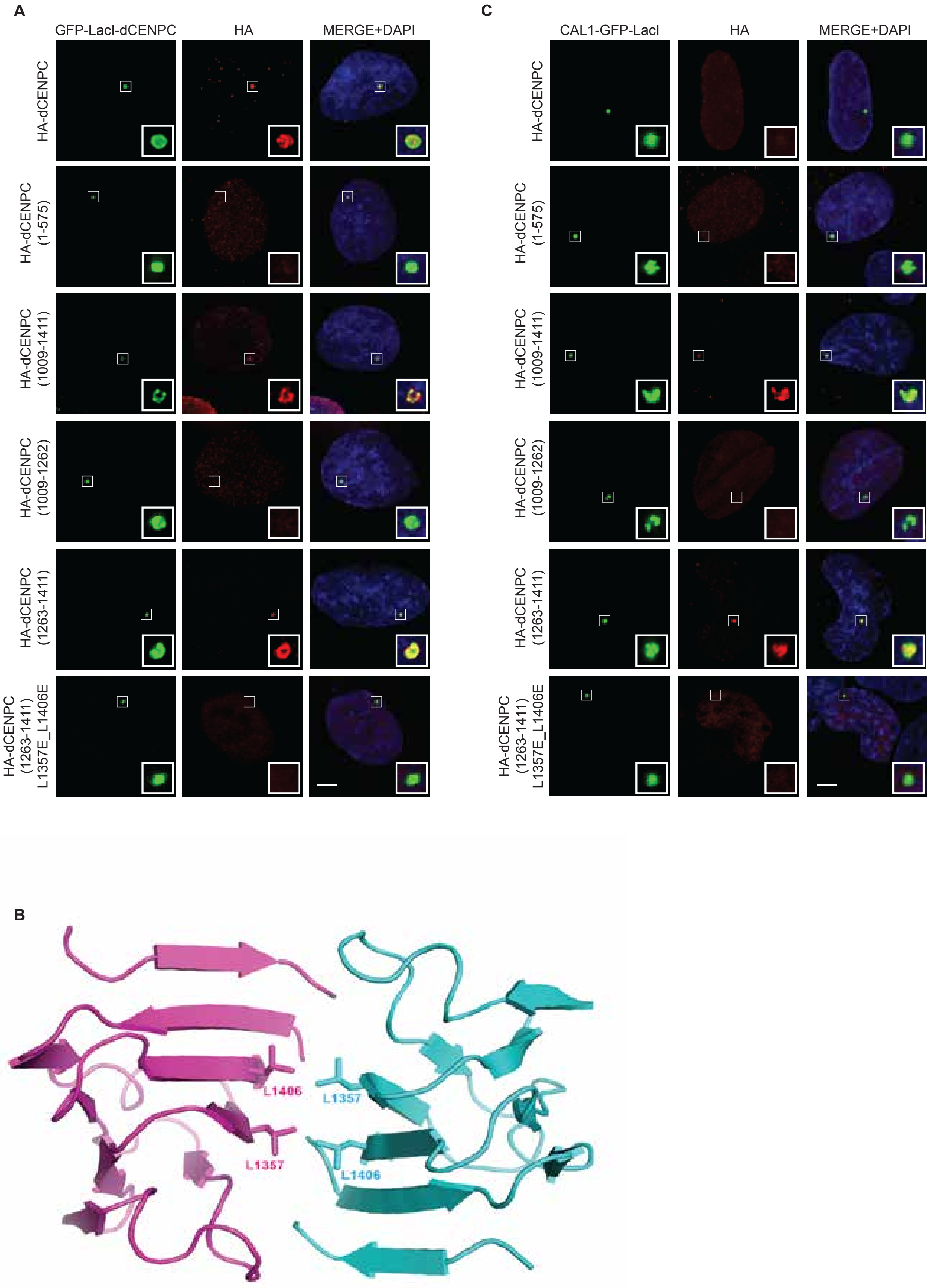

A
GFP-LacI-dCENPC
HA
MERGE+DAPI
HA-dCENPC
HA-dCENPC (1-575)
HA-dCENPC (1009-1411)
HA-dCENPC (1009-1262)
HA-dCENPC (1263-1411)
HA-dCENPC (1263-1411) L1357E_L1406E
C
CAL1-GFP-LacI
HA
MERGE+DAPI
HA-dCENPC
HA-dCENPC (1-575)
HA-dCENPC (1009-1411)
HA-dCENPC (1009-1262)
HA-dCENPC (1263-1411)
HA-dCENPC (1263-1411) L1357E_L1406E
B
L1406
L1357
L1357
L1406

Figure S6. dCENP-C dimerization is necessary for its interaction with CAL1, Related to Figure 6.

(A, C) Representative IF images of HA-tagged dCENP-C and dCENP-C fragment recruitment to the LacO arrays by GFP-LacI-dCENP-C (A) and CAL1-GFP-LacI (C) in U2OS cells. (B) Predicted crystal structure of dCENP-C(1302-1409) using Phyre2 (www.sbg.bio.ic.ac.uk/~phyre2/) (Jefferys et al., 2010; Soding, 2005).

Roure_FigS7

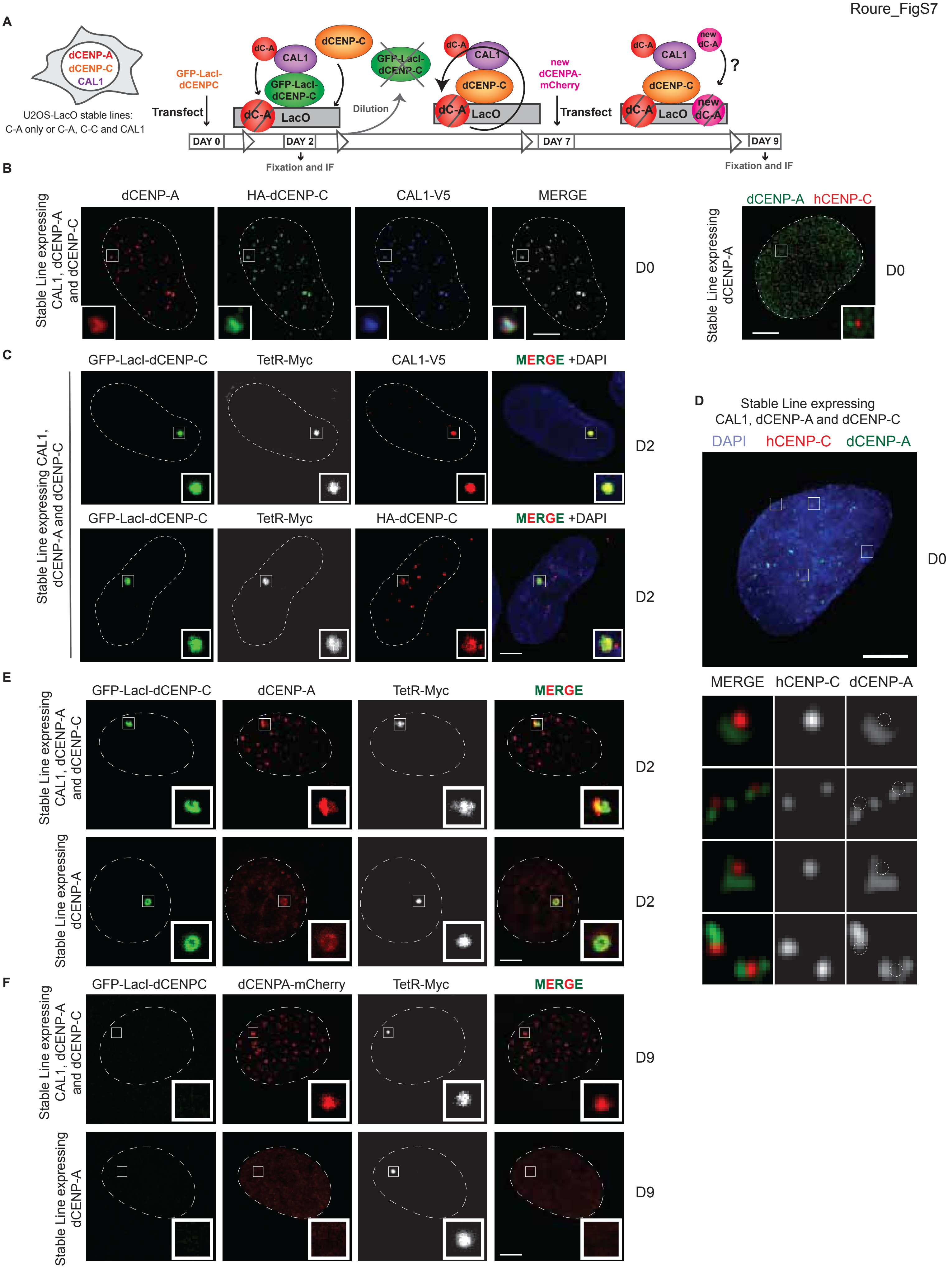
A
dCENP-A
dCENP-C
CAL1
U2OS-LacO stable lines:
C-A only or C-A, C-C and CAL1
GFP-LacI-
dCENPC
Transfect
dC-A
CAL1
dCENP-C
GFP-LacI-
dCENP-C
LacO
Dilution
new
dCENPA-
mCherry
Transfect
new
dC-A
?
DAY 0
DAY 2
DAY 7
DAY 9
Fixation and IF
Fixation and IF
B
dCENP-A
HA-dCENP-C
CAL1-V5
MERGE
Stable Line expressing CAL1, dCENP-A and dCENP-C
D0
dCENP-A hCENP-C
Stable Line expressing dCENP-A
D0
C
GFP-LacI-dCENP-C
TetR-Myc
CAL1-V5
MERGE +DAPI
D2
GFP-LacI-dCENP-C
TetR-Myc
HA-dCENP-C
MERGE +DAPI
D2
Stable Line expressing CAL1, dCENP-A and dCENP-C
D
Stable Line expressing
CAL1, dCENP-A and dCENP-C
DAPI hCENP-C dCENP-A
D0
MERGE
hCENP-C
dCENP-A
E
GFP-LacI-dCENP-C
dCENP-A
TetR-Myc
MERGE
Stable Line expressing CAL1, dCENP-A and dCENP-C
D2
Stable Line expressing dCENP-A
D2
F
GFP-LacI-dCENPC
dCENPA-mCherry
TetR-Myc
MERGE
Stable Line expressing CAL1, dCENP-A and dCENP-C
D9
Stable Line expressing dCENP-A
D9

Figure S7. dCENP-A can be stably inherited at ectopic chromosomal sites where *Drosophila* centromere factors are artificially targeted, Related to Figure 7.

(A) Experimental procedure for the dCENP-A propagation experiments using newly transfected dCENP-A-mCherry. (B) IF images of U2OS-LacO stable cell lines expressing CAL1-V5, dCENP-A and HA-dCENP-C or only dCENP-A (right image; co-stained with hCENP-C Day 0). (C) Representative IF images of CAL1-V5 and HA-dCENP-C recruitment to the LacO arrays 2 days (D2) after transfection of GFP-LacI-dCENP-C in U2OS-LacO stable cell line expressing the 3 centromere proteins. (D) Triple transgene expressing stable line stained for dCENP-A and hCENP-C at Day 0 prior to transfection. (E, F) Representative IF images of the recruitment to the LacO arrays of dCENP-A 2 days (D2) after transfection of GFP-LacI-dCENP-C (E) and dCENP-A-mCherry (F) 9 days (D9) after transfection of GFP-LacI-dCENP-C in U2OS-LacO stable cell line expressing the 3 centromere proteins or only dCENP-A. All insets show magnification of the boxed regions. Scale bar, 5μm.
